# Supplementary material for: A KLF6-driven transcriptional network links lipid homeostasis and tumour growth in renal carcinoma
Source: Nat Commun. 2019 Mar 11;10:1152. doi: 10.1038/s41467-019-09116-x (PMC6411998; doi:10.1038/s41467-019-09116-x)
Supplement: Supplementary file 1 — Supplementary Information [file 41467_2019_9116_MOESM1_ESM.pdf]

A KLF6-driven transcriptional network links lipid homeostasis and tumour growth in renal carcinoma

Syafruddin et al.

Supplementary Information

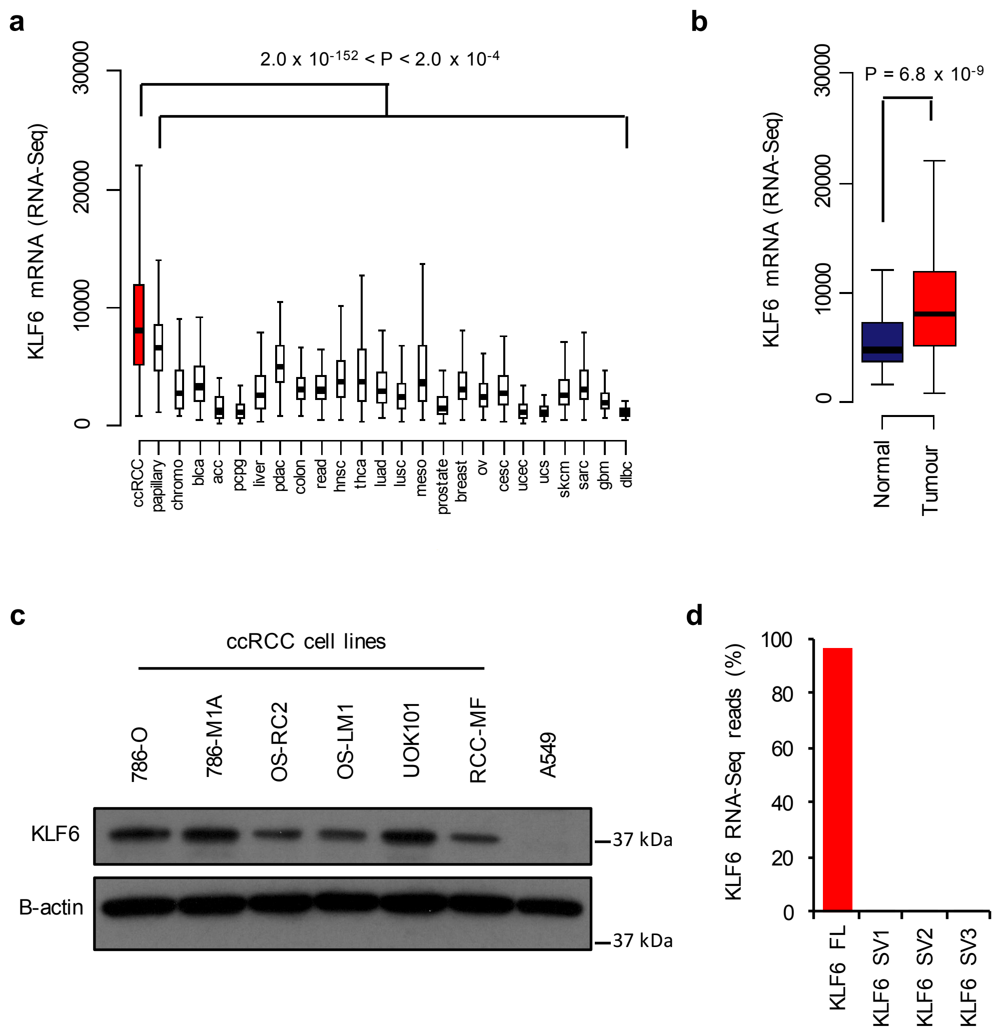

Supplementary Figure 1. Full length *KLF6* is highly expressed in ccRCC. (a) *KLF6* expression (RSEM normalized counts) in ccRCC and other tumour types from the TCGA cohort. Mann Whitney U test. **(b)** *KLF6* expression (RSEM normalized counts) in ccRCC and normal kidney tissues in the TCGA cohort. Mann Whitney U test. **(c)** KLF6 protein expression in a panel of ccRCC cell lines and A549, a lung cancer cell line. **(d)** Transcript reads of the full length and alternatively spliced *KLF6* variants as measured by RNA-seq in ccRCC cell lines. Boxplots represent median and 25th and 75th percentiles, whiskers extend to data extremes.

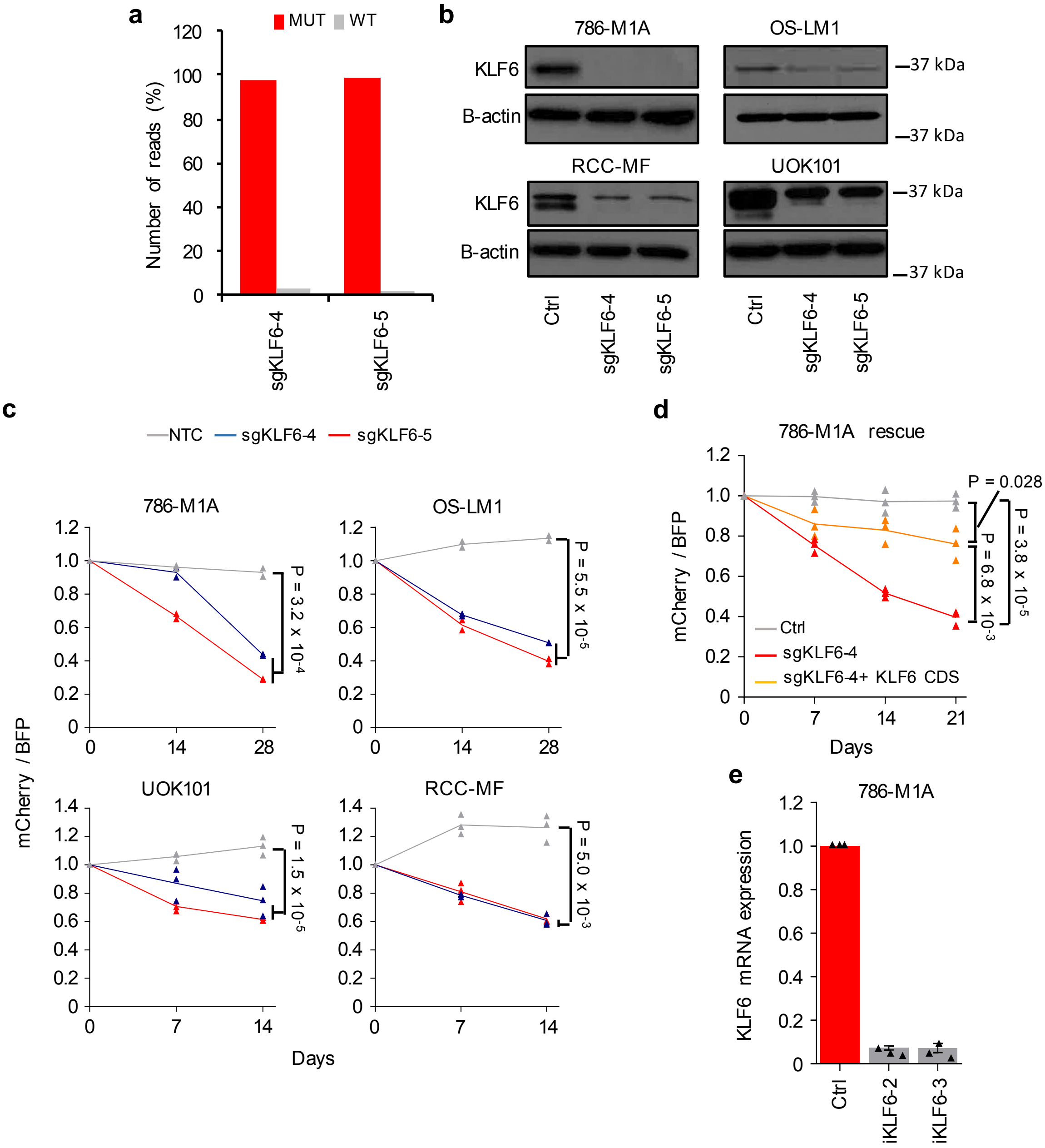

Supplementary Figure 2. CRISPR-Cas9-mediated KLF6 targeting impairs ccRCC cell growth in vitro. (a) Fraction of wild type and mutant KLF6 genomic DNA in cells targeted by CRISPR-Cas9 using sgKLF6-4 and sgKLF6-5. **(b)** KLF6 immunoblot in pools of cells transduced with CRISPR-Cas9 sgKLF6-4, sgKLF6-5, and non-targeting control. **(c)** Competitive proliferation assay of KLF6-targeted ccRCC cells (pools of lentivirally transduced CRISPR-Cas9 knock-out cells). Relative fraction of $mCherry^+$ KLF6-targeted and BFP^+ control cells, normalized to day 0. 786-M1A and OS-LM1, average of two technical replicates; UOK101 and RCC-MF average of three technical replicates. Two-tailed Student's t-test. **(d)** (Top) Competitive proliferation assay of CRISPR-Cas9 KLF6-targeted 786-M1A cell pools transduced with exogenous KLF6. Relative fraction of $mCherry^+$ sgKLF6-4 or sgKLF6-4 with exogenous KLF6, normalized to day 0, compared to BFP^+ control cells. Average of three technical replicates. Two-tailed Student's t-test. **(e)** KLF6 mRNA expression, as measured by qRT-PCR, in the KLF6-targeted 786-M1A CRISPRi cells. Average of three experiments. Error bars, SEM.

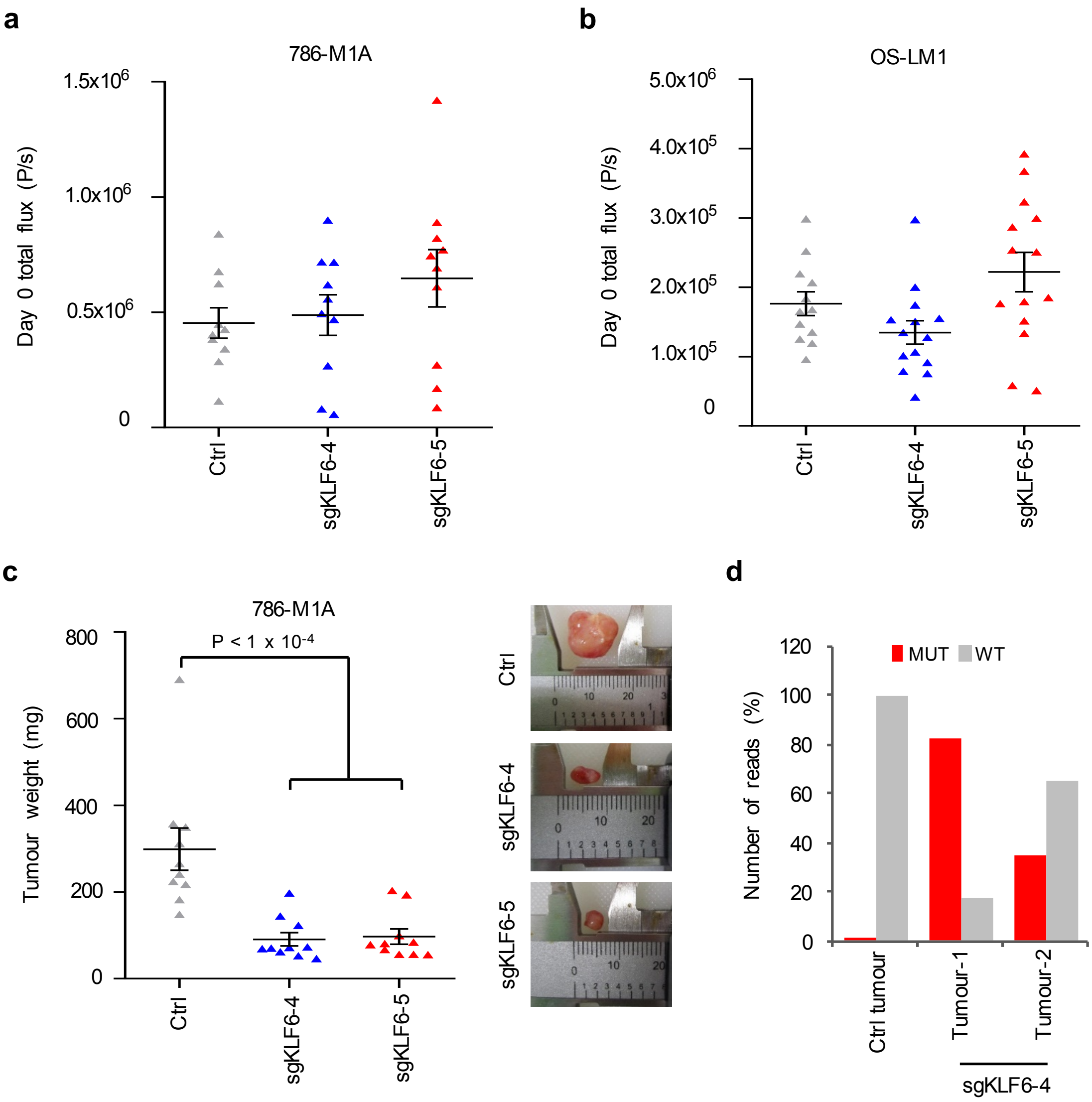

Supplementary Figure 3. Inhibition of tumour growth by CRISPR-Cas9-mediated KLF6 targeting. (a, b) Bioluminescence signal of the KLF6-targeted and control 786-M1A **(a)** and OS-LM1 **(b)** cells at day 0 of the experiments shown in Figure 2a-b. **(c)** Tumor weights at the end of the experiment shown in Figure 2a. Two-tailed Mann-Whitney U test. **(d)** Fraction of wild type and mutant KLF6 genomic DNA in the control and sgKLF6-4 subcutaneous tumours.

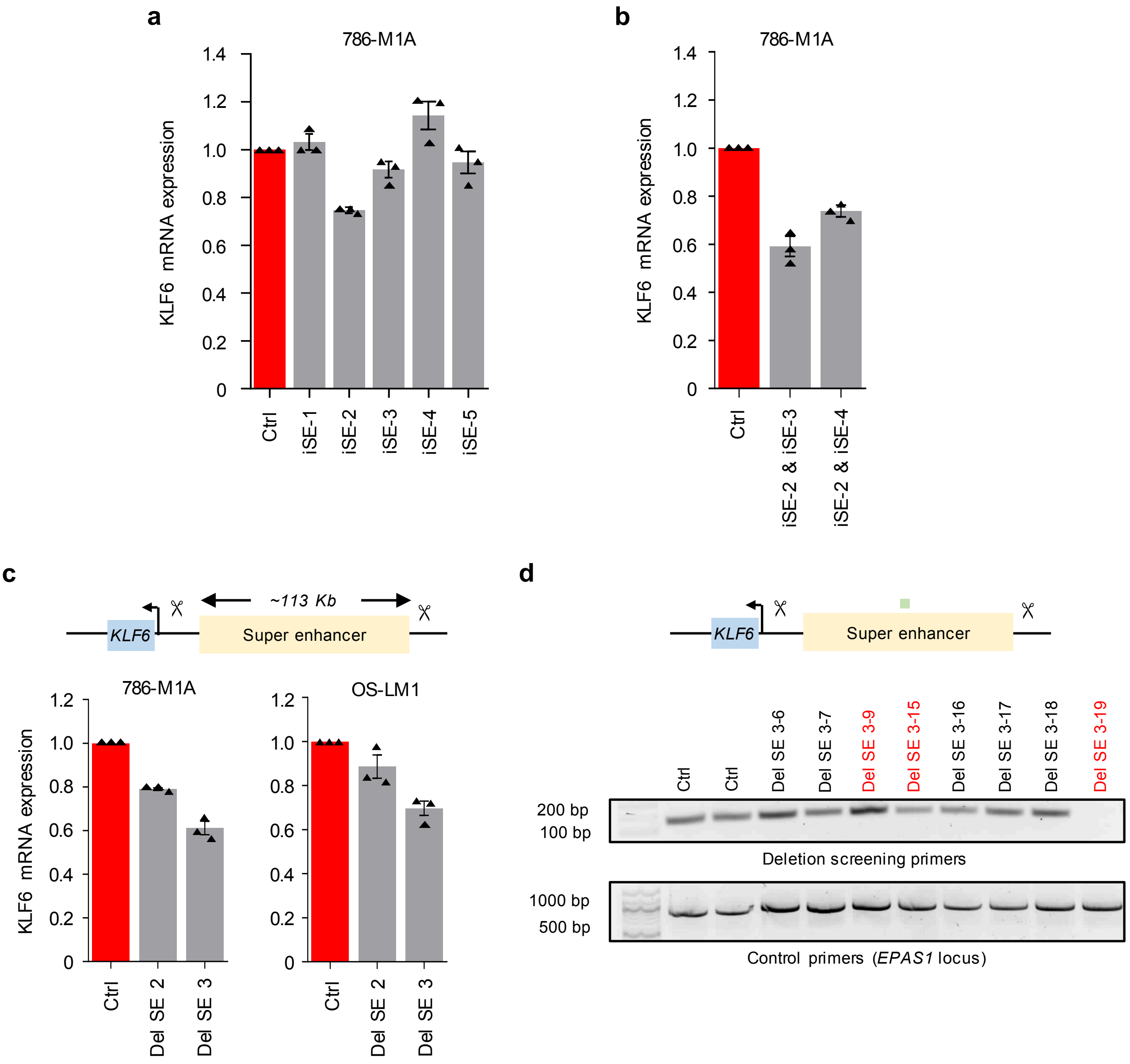

Supplementary Figure 4. A robust super enhancer regulates *KLF6* expression in ccRCC. (a, b) KLF6 mRNA expression in each of the enhancer-targeted cells shown in figure 3a (a) and in cells with combinatorial enhancer targeting (b). Average of three experiments. Error bars, SEM. **(c)** (Top) Strategy for CRISPR-Cas9-mediated deletion of the large super enhancer region. (Bottom) KLF6 mRNA expression in the CRISPR-Cas9 super enhancer-deleted population of 786-M1A and OS-LM1 cells. Average of three experiments. Error bars, SEM. **(d)** PCR-based screening for single cell-derived clones that harbor deletions of the super enhancer region. Clones shown in red, Del SE 3-9 (no deletion), Del SE 3-15 (putative heterozygous deletion), and Del SE 3-19 (putative homozygous deletion) were selected for further analysis. Green bar shows the location of the SE PCR target region, the *EPAS1* locus was used as a genomic control region.

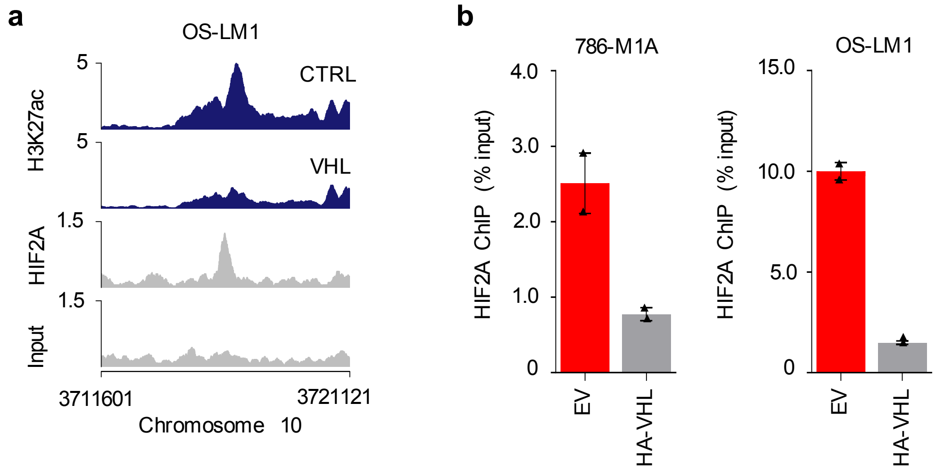

Supplementary Figure 5. HIF2A binds the *KLF6* enhancer region. (a) A close up of the region highlighted in Figure 4d for OS-LM1 cells together with HIF2A ChIP-seq signal in the parental OS-LM1 cells. **(b)** ChIP-qPCR targeting the HIF2A peak shown in panel a. Average of two experiments. Error bars, SEM.

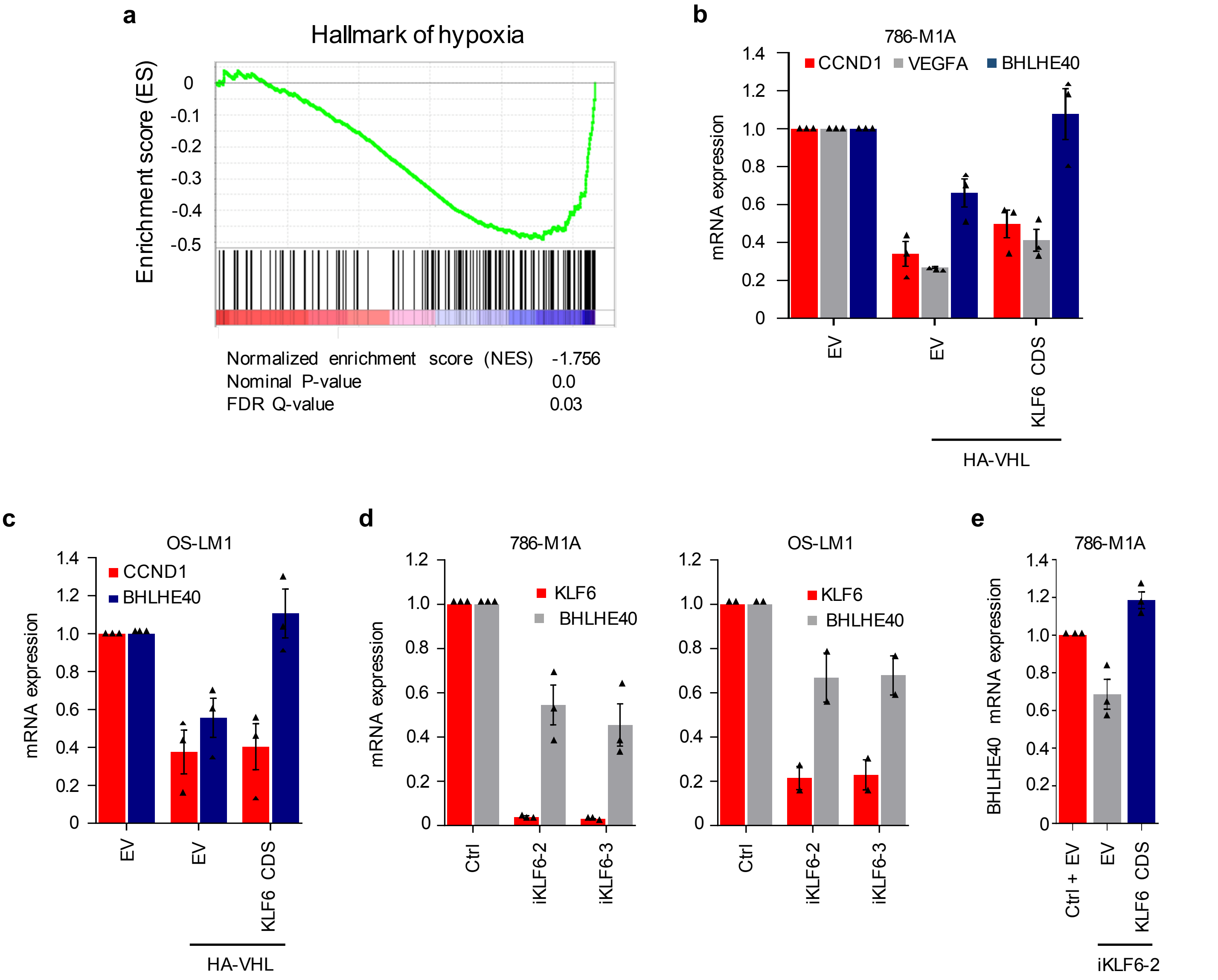

Supplementary Figure 6. KLF6 modulates the expression of *BHLHE40,* a HIF2A downstream target. (a) Gene set enrichment analysis shows downregulation of the canonical hypoxia-response gene set in the KLF6-depleted CRISPRi cells. Red, high correlation; blue, low correlation. **(b)** Expression of HIF2A downstream targets *CCND1*, *VEGFA* and *BHLHE40* in the VHL-restored 786-M1A cells, reintroduced with either EV or exogenous KLF6. Average of three experiments. Error bars, SEM. **(c)** Expression of HIF2A downstream targets *CCND1* and *BHLHE40* in the VHL-restored OS-LM1 cells, reintroduced with either EV or exogenous KLF6. Average of three experiments. Error bars, SEM. **(d)** Expression of *KLF6* and *BHLHE40* in KLF6-depleted CRISPRi cells. Average of three experiments for 786-M1A cells, average of two experiments for OS-LM1 cells. Error bars, SEM. **(e)** *BHLHE40* expression in in KLF6-depleted 786-M1A CRISPRi cells reintroduced with exogenous KLF6. Average of three experiments. Error bars, SEM.

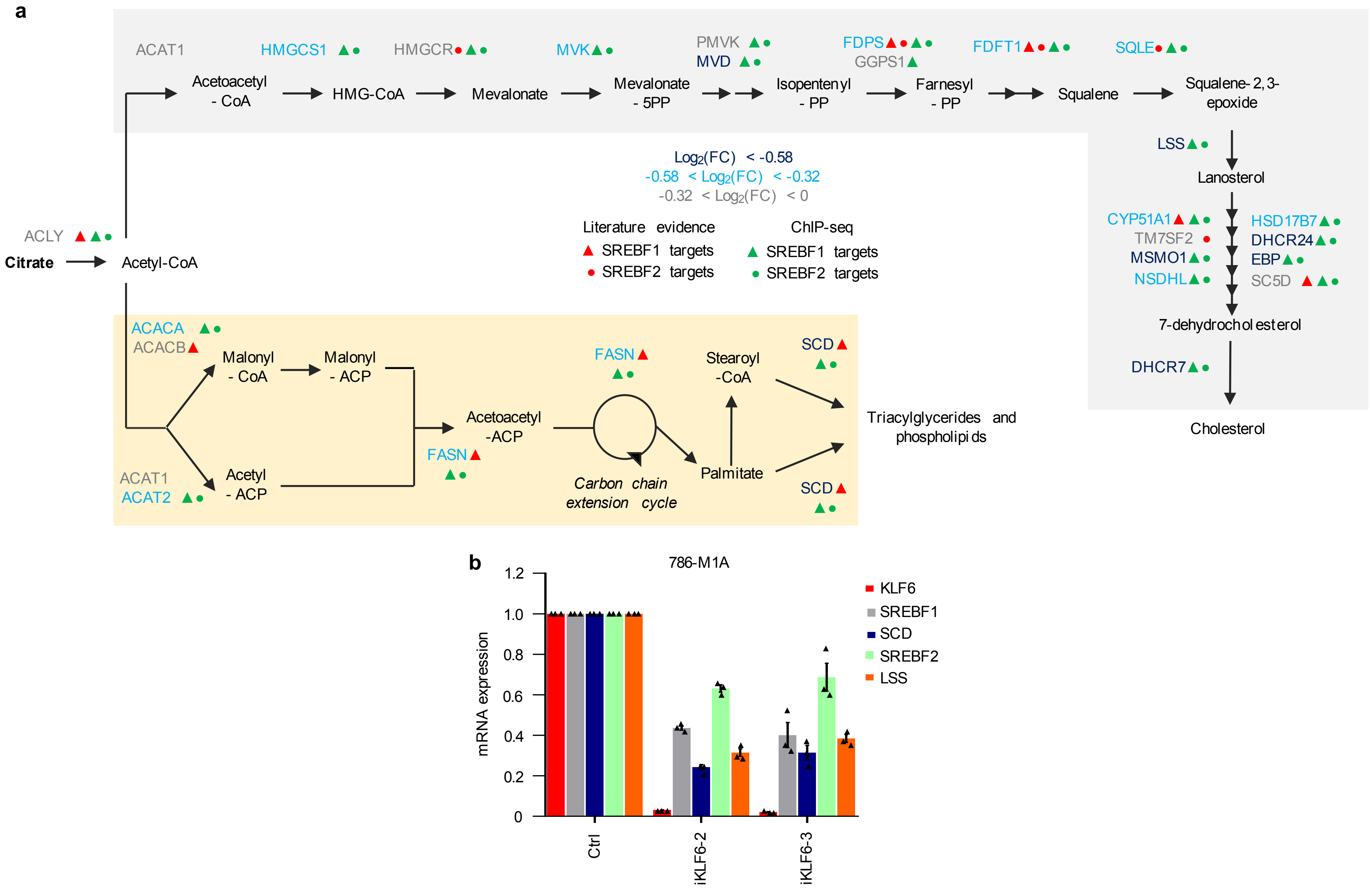

Supplementary figure 7. Coordinated downregulation of lipid homeostasis-associated genes upon KLF6 inhibition. (a) A simplified schematic of triacylglycerol and cholesterol biosynthesis pathways. Colour coding represents fold change in CRISPRi KLF6 targeted cells. SREBF1 and SREBF2 target genes determined by systematic literature analysis (red triangles and circles) and ChIP-seq analysis (green triangles and circles). **(b)** Validation of RNA-seq data using qRT-PCR. Average of three experiments. Error bars, SEM.

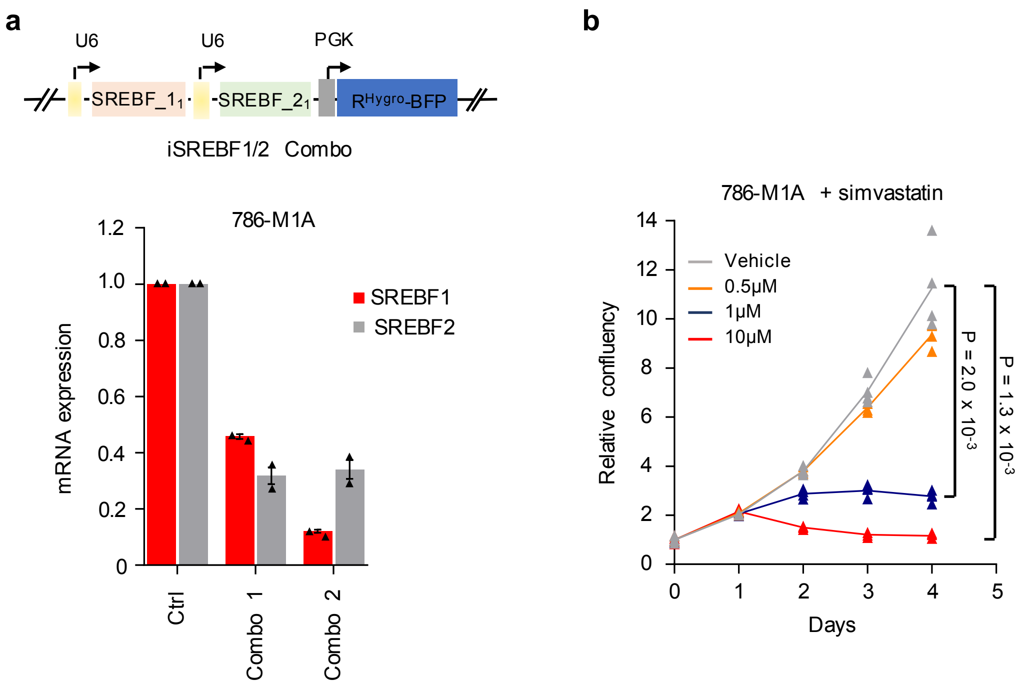

Supplementary Figure 8. Inhibition of cholesterol biosynthesis impairs ccRCC growth in vitro. (a) (Top) Illustration of the construct used for combinatorial targeting of SREBF1 and SREBF2 by CRISPRi. (Bottom) *SREBF1* and *SREBF2* expression in SREBF1 and SREBF2-targeted 786-M1A CRISPRi cells. Two independent combinatorial targeting constructs used (Combo 1 and Combo 2). Average of two experiments. Error bars, SEM. **(b)** Relative confluency of the 786-M1A cells treated with either vehicle or the indicated concentrations of simvastatin. Average of four technical replicates. Two-tailed Student's t-test.

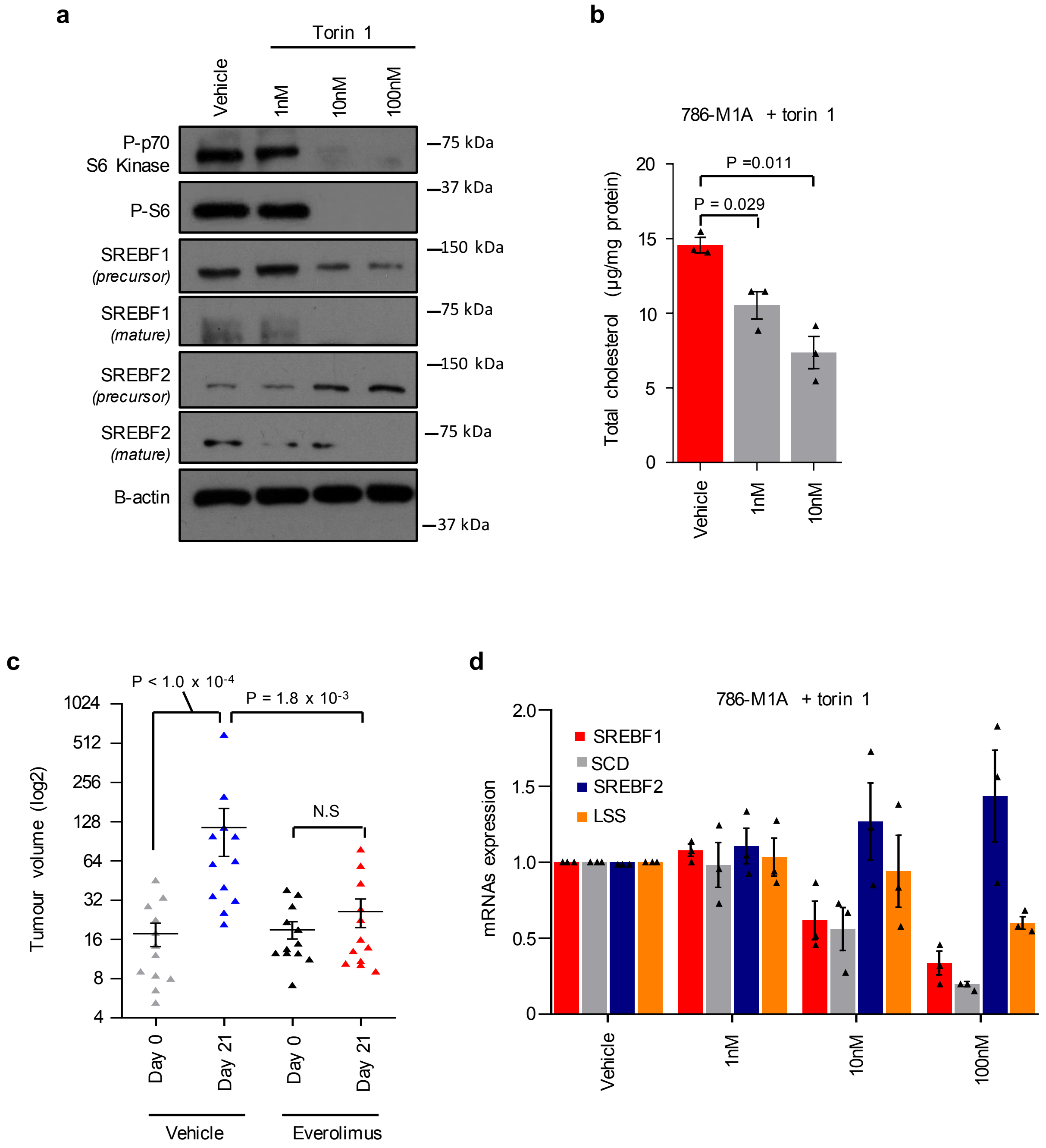

Supplementary Figure 9. mTORC1 complex modulates SREBF1/2 expression and activity in ccRCC. (a) mTORC1 and SREBF1/2 activity in the 786-M1A cells as determined by immunoblotting after torin 1 treatment for 48 hours. Representative of two experiments are shown. **(b)** Total cholesterol level in the 786-M1A cells treated with torin 1 for 48 hours. Average of three experiments. Error bars, SEM. Two-tailed Student's t-test. **(c)** Tumour volume from 786-M1A cells in mice fed with everolimus or vehicle pre- and 21 days post-treatment. N=12 tumours/group. Two-tailed Mann-Whitney U test. **(d)** Expression of *SREBF1*, *SCD*, *SREBF2* and *LSS* in 786-M1A cells treated with torin 1 for 48 hours as measured by qRT-PCR. Average of three experiments. Error bars, SEM.

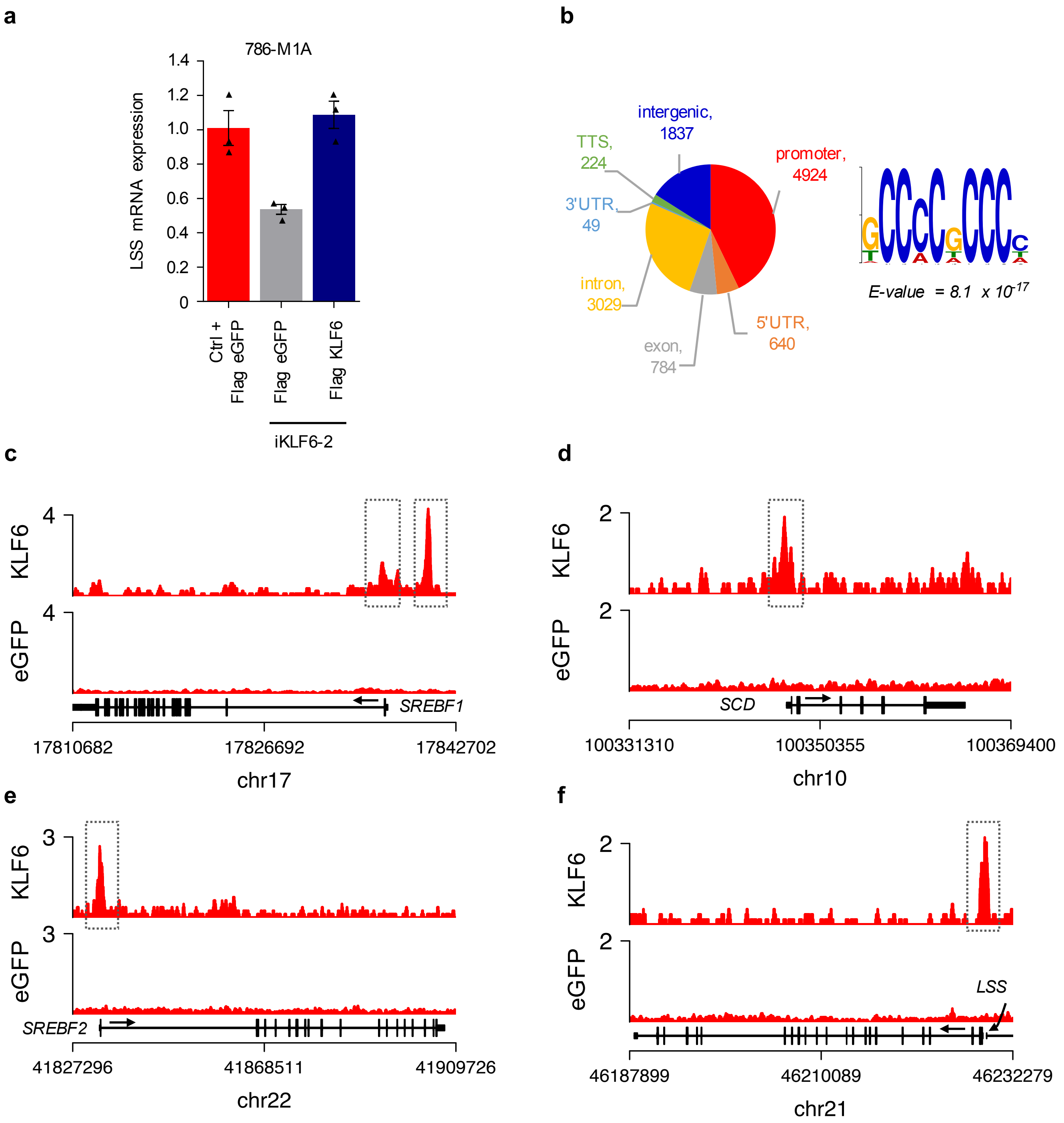

Supplementary Figure 10. KLF6 binds *SREBF1, SREBF2, SCD* and *LSS* regulatory regions. (a) *LSS* expression in the KLF6-targeted 786-M1A CRISPRi cells, transduced with either exogenous flag-eGFP or flag-KLF6. Average of three technical replicates is shown. Error bars, SEM. **(b)** Left, Genomic distribution of the 11487 KLF6 peaks relative to known transcripts. Right, the most significant DNA motif detected using MEME de novo motif discovery on the 500 most significant KLF6 peaks. **(c-f)** Flag ChIP-seq signal in the genomic regions of *SREBF1* (c), *SCD* (d), *SREBF2* (e) and *LSS* (f). Arrows indicate the direction of transcription, dashed boxes indicate KLF6 peaks.

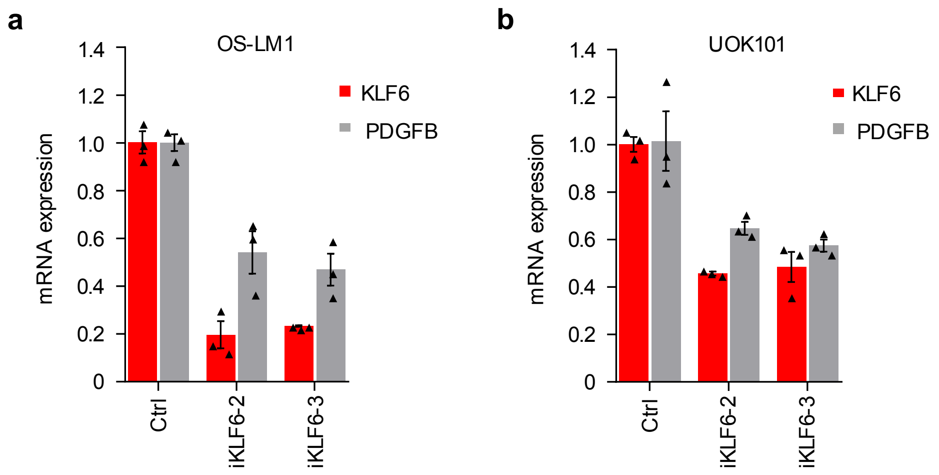

Supplementary Figure 11. Downregulation of *PDGFB* expression in the KLF6-targeted cells. (a, b) *PDGFB* expression in KLF6-targeted OS-LM1 (a) and UOK101 (b) CRISPRi cells, as measured by qRT-PCR. Three technical replicates. Error bars, SEM.

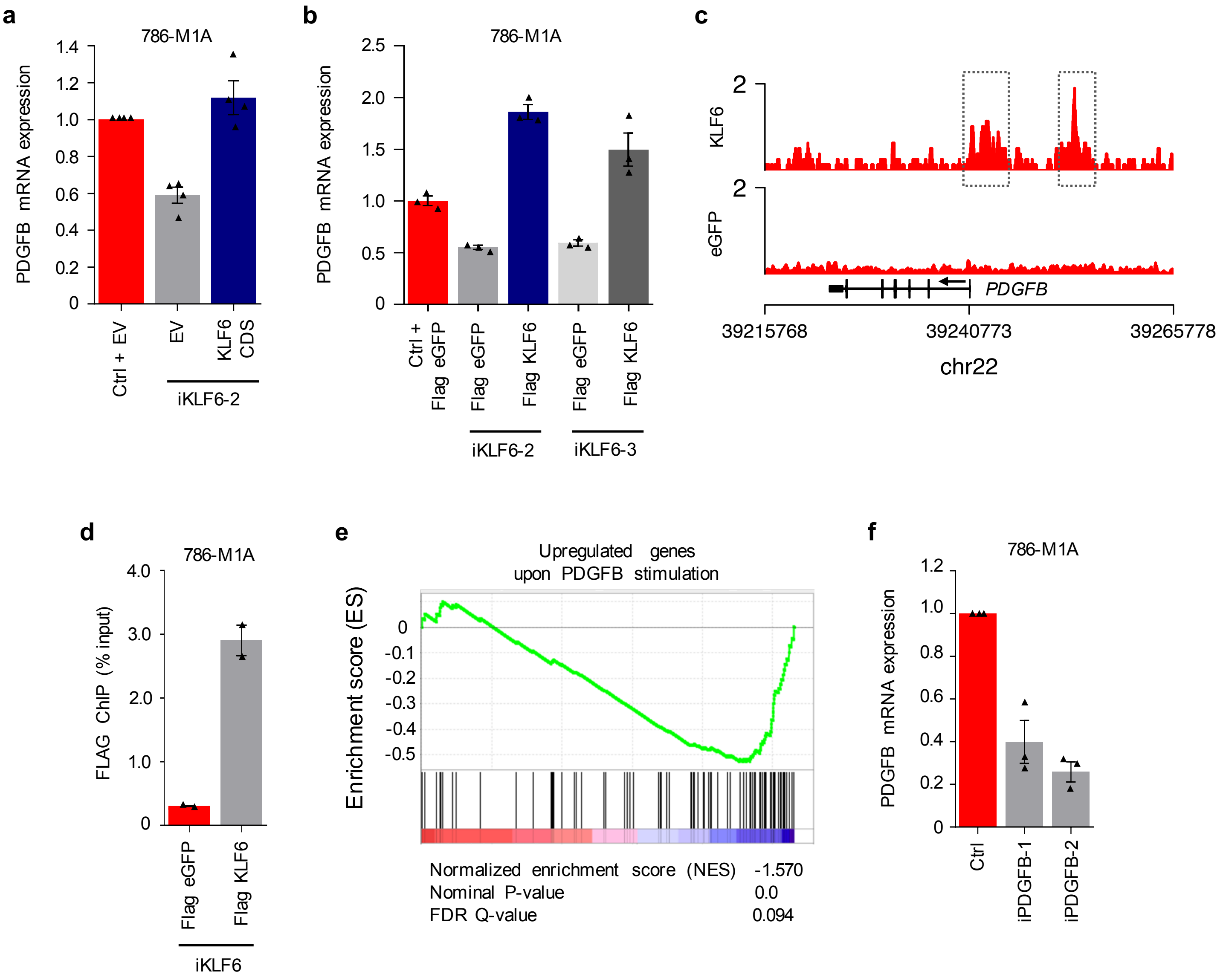

Supplementary Figure 12. KLF6 regulates *PDGFB* expression in ccRCC. (a) *PDGFB* expression in the pool of KLF6-targeted 786-M1A CRISPRi cells, transduced with either an empty vector or exogenous KLF6. Average of four experiments. Error bars, SEM. **(b)** *PDGFB* expression in the pool KLF6-targeted 786-M1A CRISPRi cells, transduced with either exogenous Flag eGFP or Flag KLF6. The average of three technical replicates is shown. Error bars, SEM. **(c)** Flag ChIP-seq signal in the *PDGFB* locus. Arrow indicates the direction of transcription, dashed box indicates KLF6 peaks. **(d)** Flag ChIP-qPCR targeting PDGFB promoter. Average of two experiments. Error bars, SEM. **(e)** Gene set enrichment analysis showing downregulation of PDGFB-responsive genes in the KLF6-targeted 786-M1A CRISPRi cells. Red, high correlation; blue, low correlation. **(f)** *PDGFB* expression in the pool of PDGFB-targeted 786-M1A CRISPRi cells. Average of three experiments. Error bars, SEM.

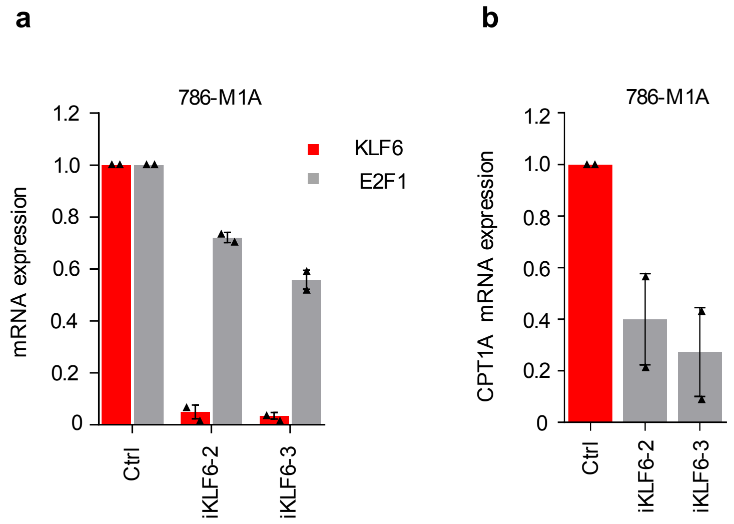

Supplementary Figure 13. *E2F1* and *CPT1A* downregulation upon KLF6 inhibition in ccRCC cells. (a) *KLF6* and *E2F1* expression in the pool of KLF6-targeted 786-M1A CRISPRi cells as measured by qRT-PCR. Average of two experiments. Error bars, SEM. **(b)** *CPT1A* expression in the pool of KLF6-targeted 786-M1A CRISPRi cells as measured by qRT-PCR. Average of two experiments. Error bars, SEM.

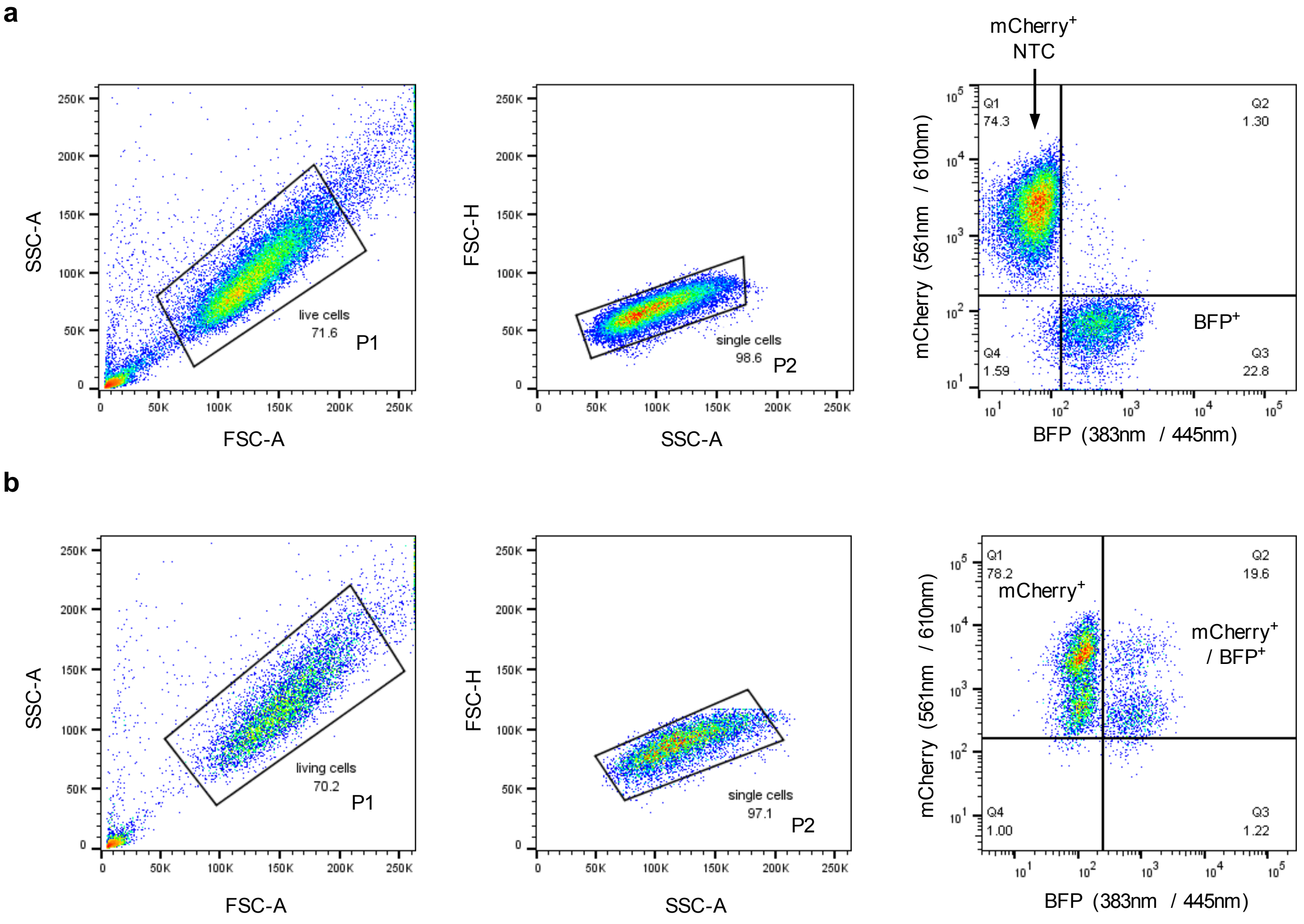

Supplementary Figure 14. Gating strategy for competitive proliferation assays. (a) Example of gating strategy for the CRISPR-Cas9 competitive proliferation assay. FACS plot of the mixed population of BFP^+ sgKLF6-5 and $mCherry^+$ NTC 786-M1A cells at day 28. **(b)** Example of gating strategy for the CRISPRi competitive proliferation assay. FACS plot of the mixed population of $mCherry^+$/ BFP^+ iKLF6-2 and $mCherry^+$ only 786-M1A CRISPRi cells at day 25.

a

Full scans for Figure 1e

KLF6

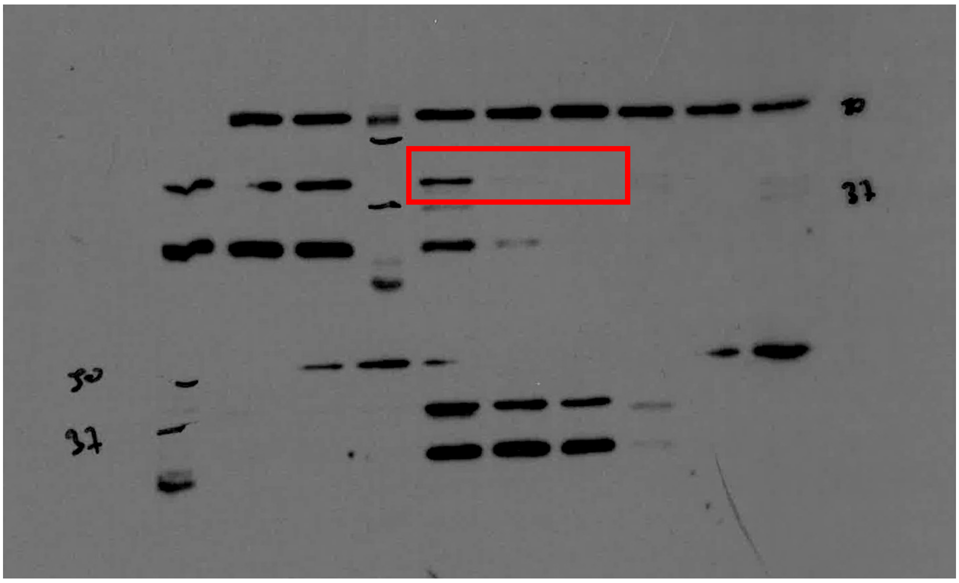

B-actin

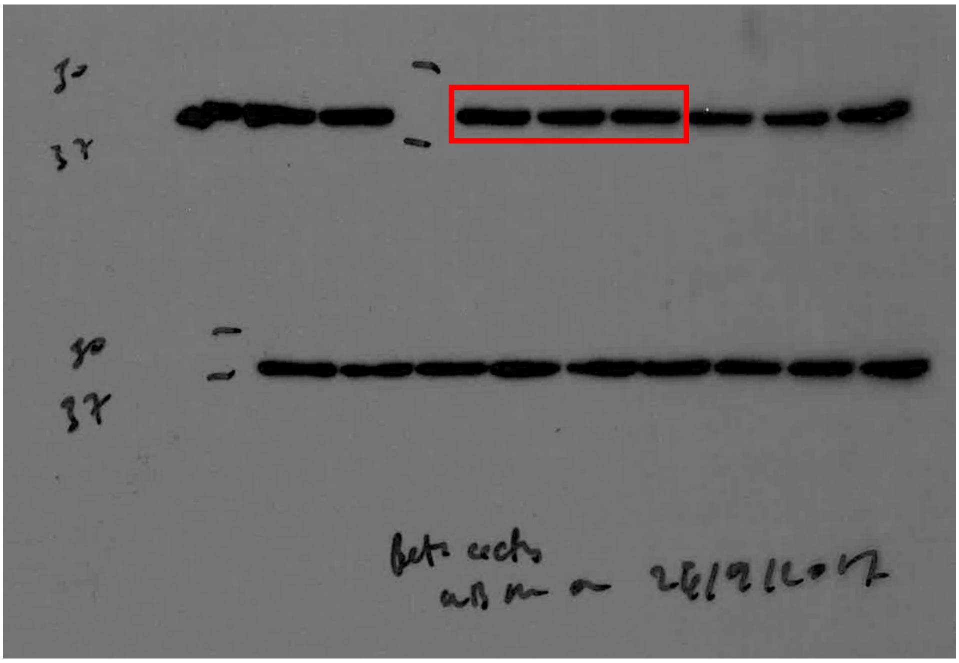

b

Full scans for Figure 1f

KLF6

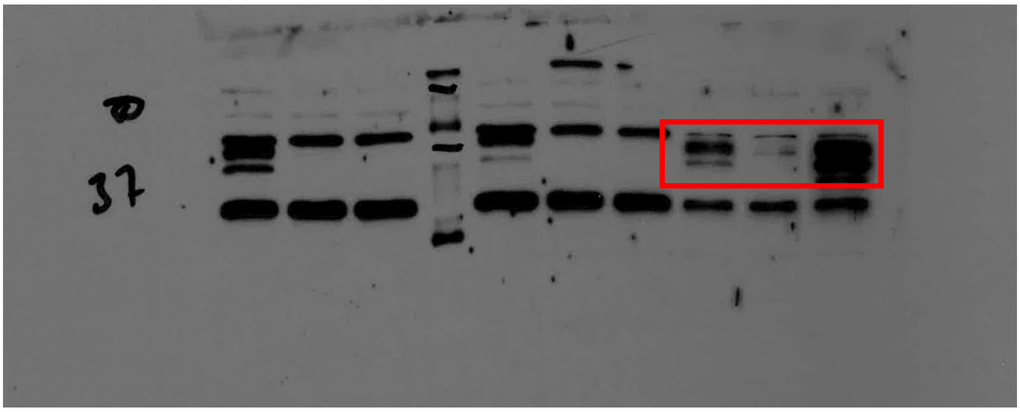

B-actin

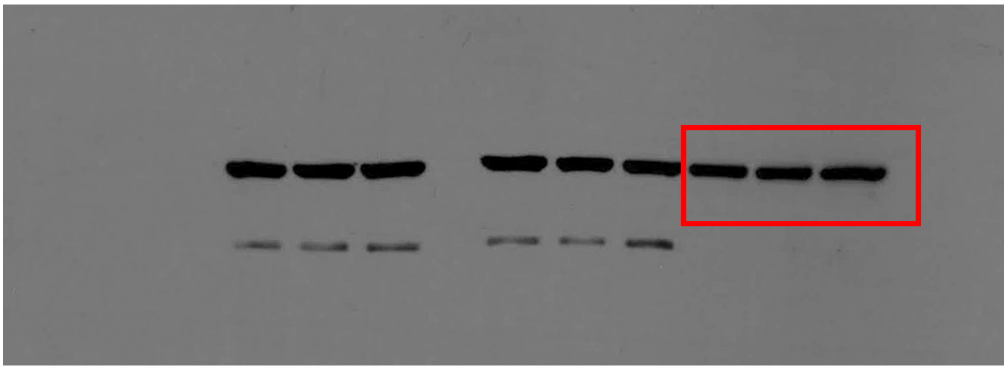

c

Full scans for Figure 4c

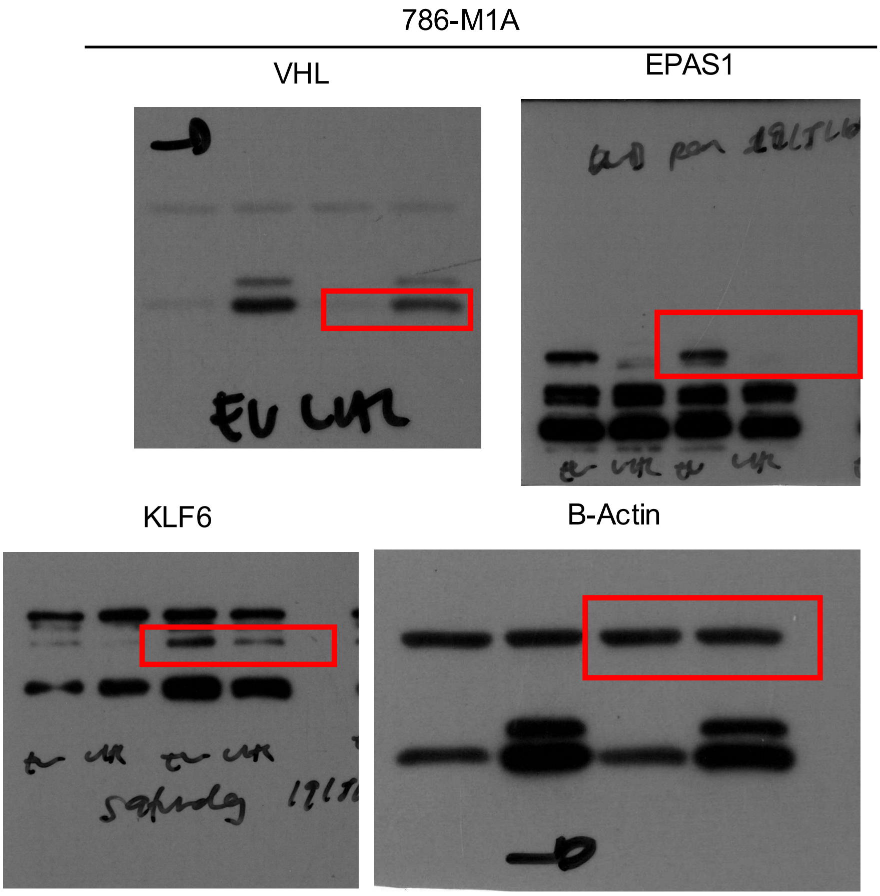

d

Full scans for Figure 4c

OS-LM1

VHL

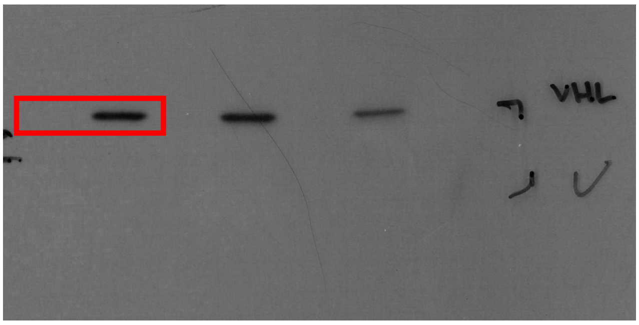

EPAS1

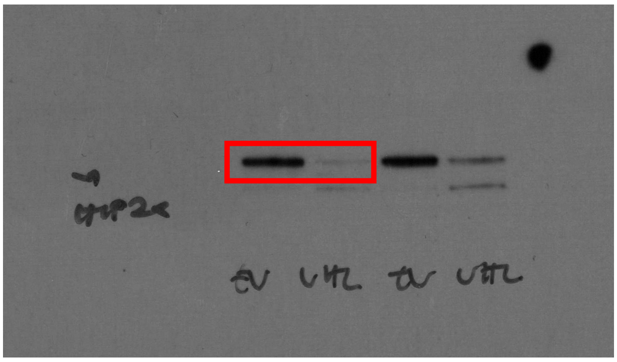

B-actin for VHL

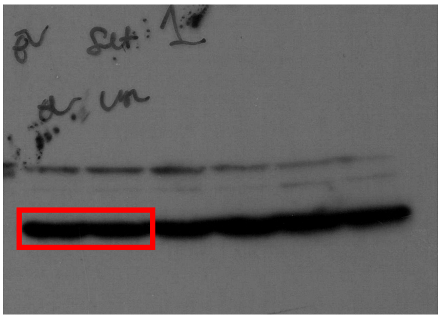

KLF6

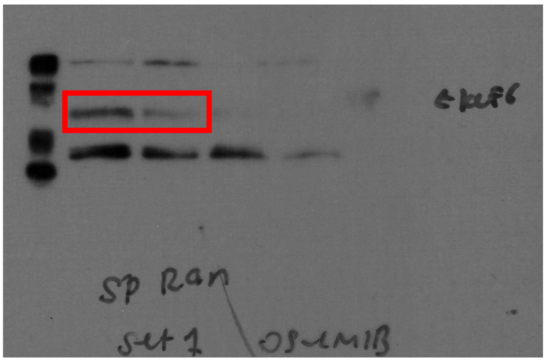

B-actin for EPAS1 and KLF6

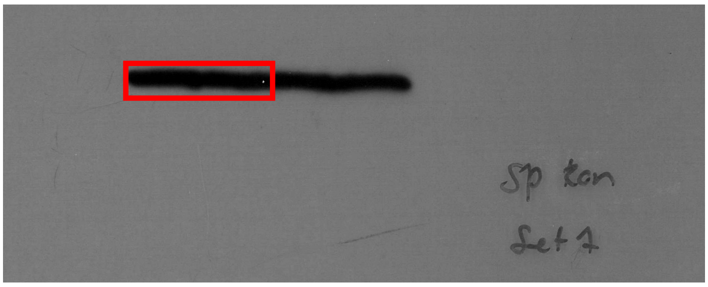

e

Full scans for Figure 6a

KLF6

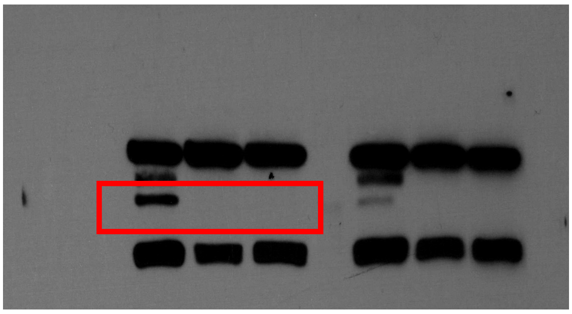

P-p70 S6 kinase

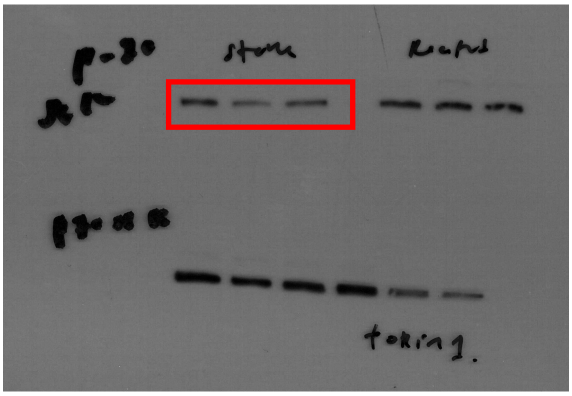

P-S6

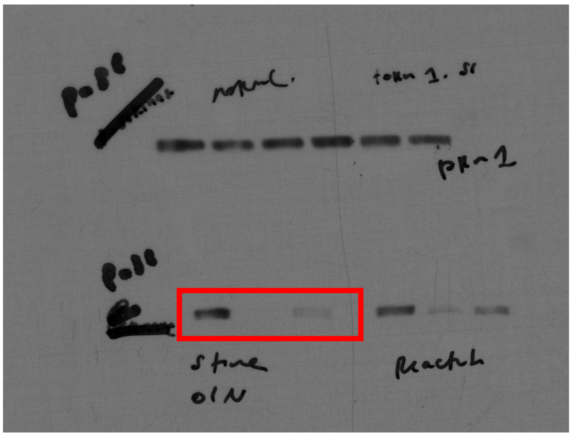

S6

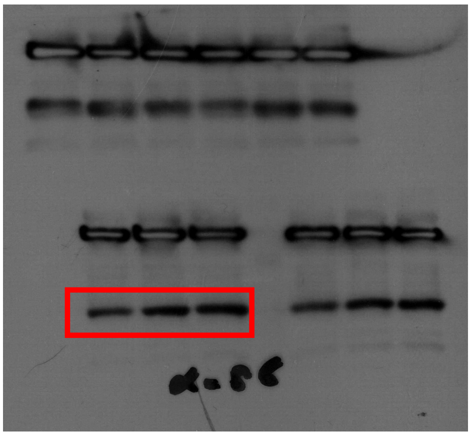

B-actin

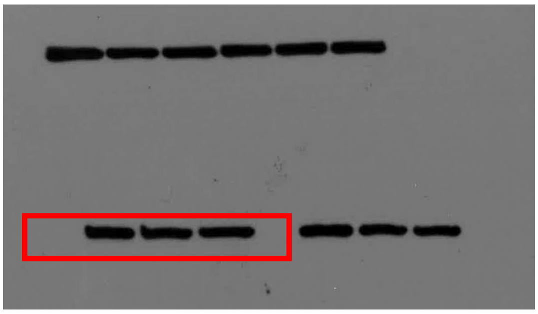

B-actin (p70 S6)

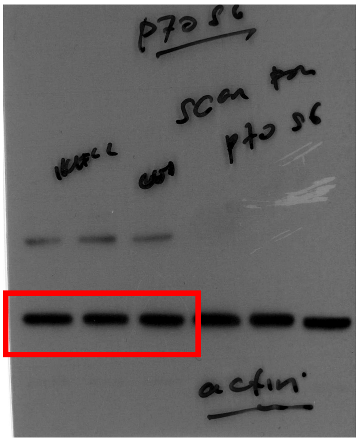

p70 S6

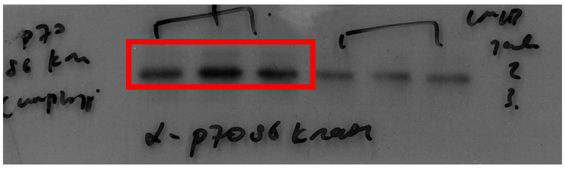

f

Full scans for Figure 6b

OS-LM1

KLF6

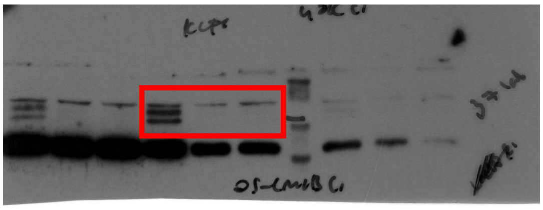

P-S6

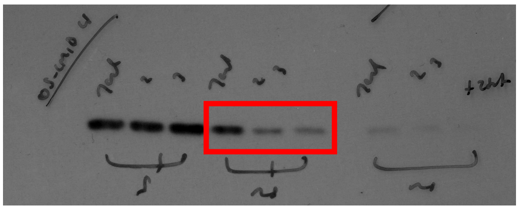

S6

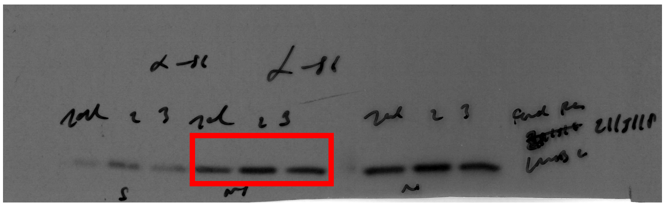

B-actin

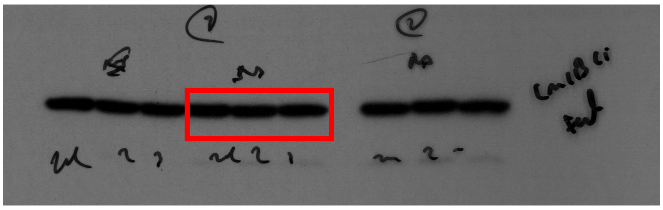

UOK101

KLF6

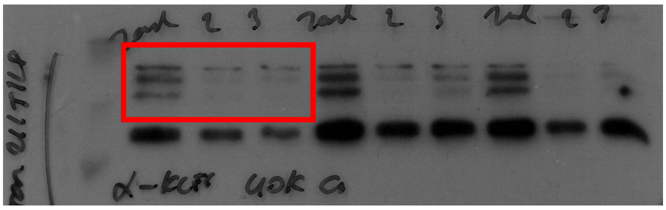

P-S6

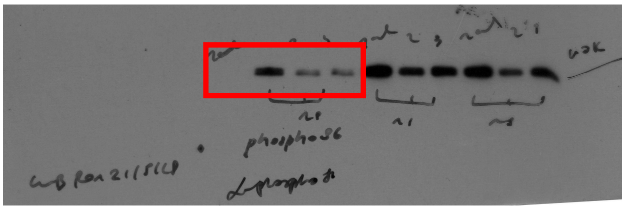

S6

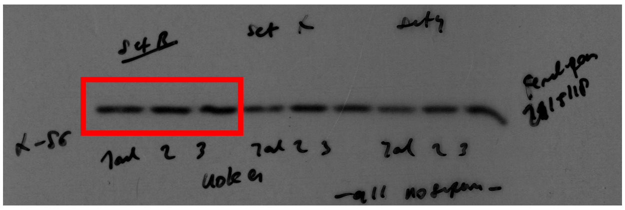

B-actin

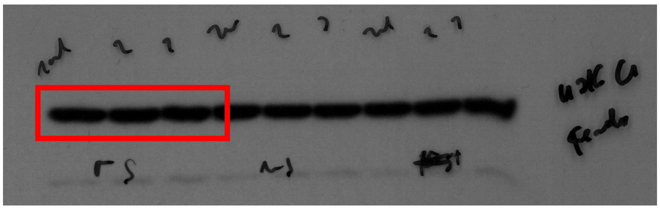

g

Full scans for Figure 6e

P-p70 S6 kinase

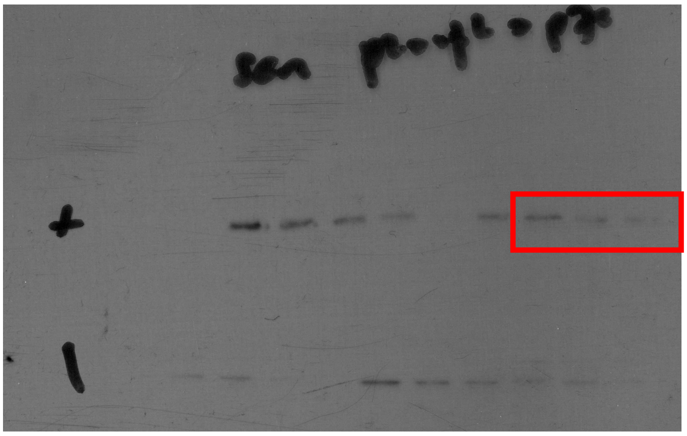

P-S6

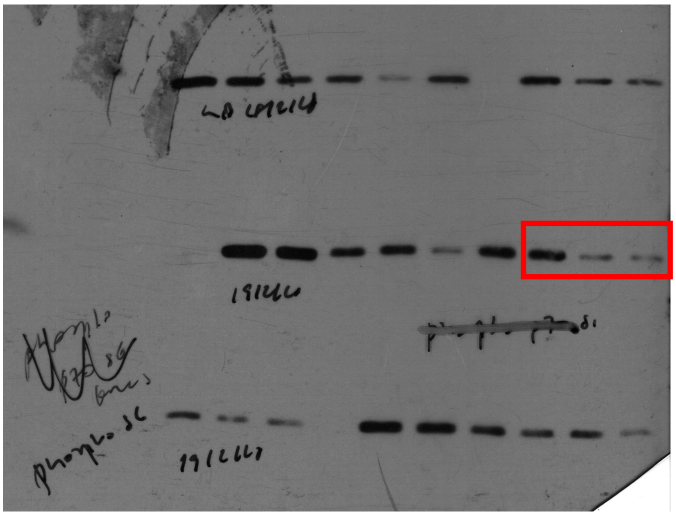

p70 S6 kinase

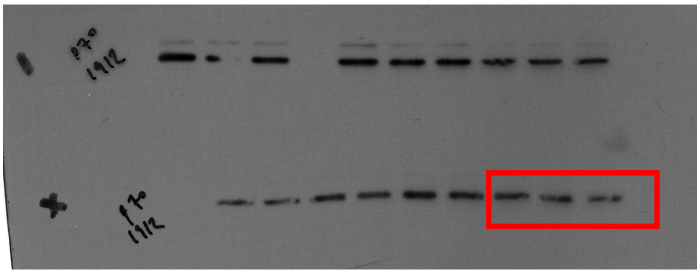

S6

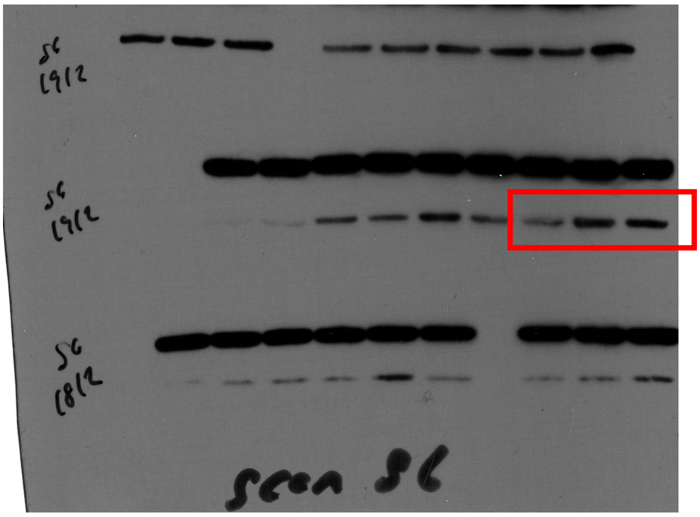

B-actin

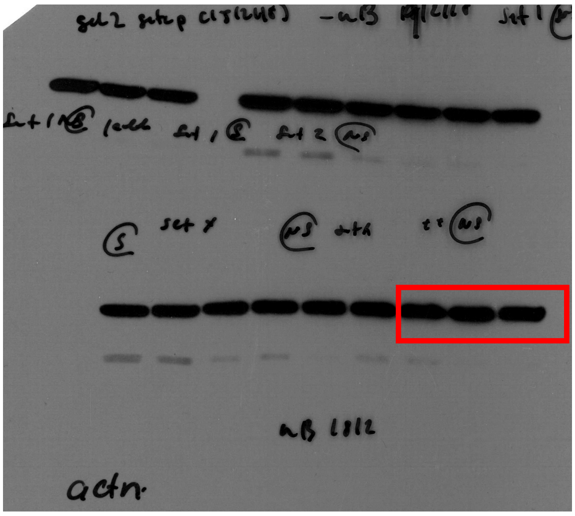

h

Full scans for Figure 6f and 6g

KLF6

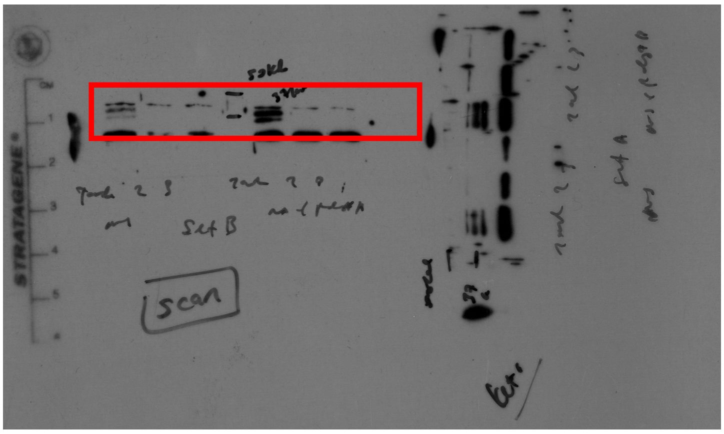

p70 S6 kinase

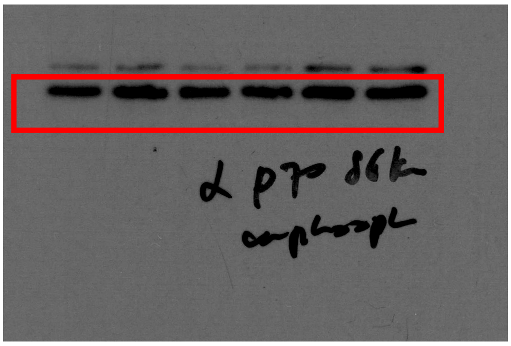

P-p70 S6 kinase

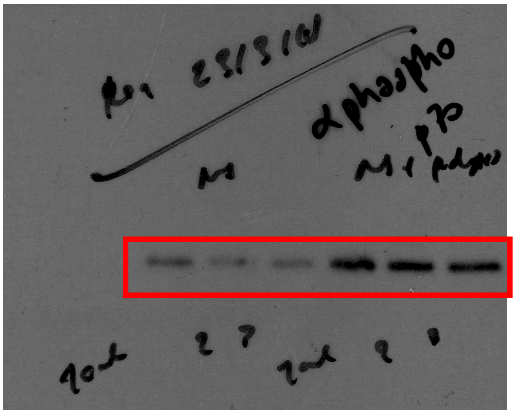

P-S6

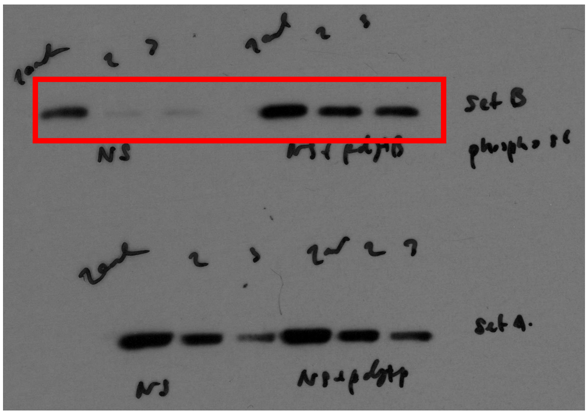

S6

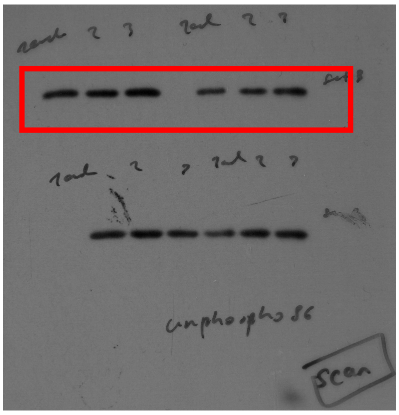

B-actin

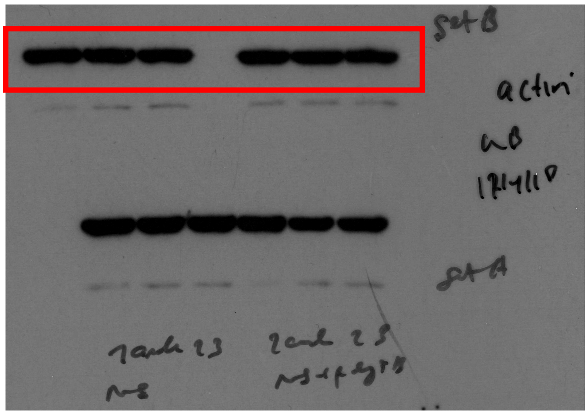

i

Full scans for Supplementary Figure 1c

KLF6

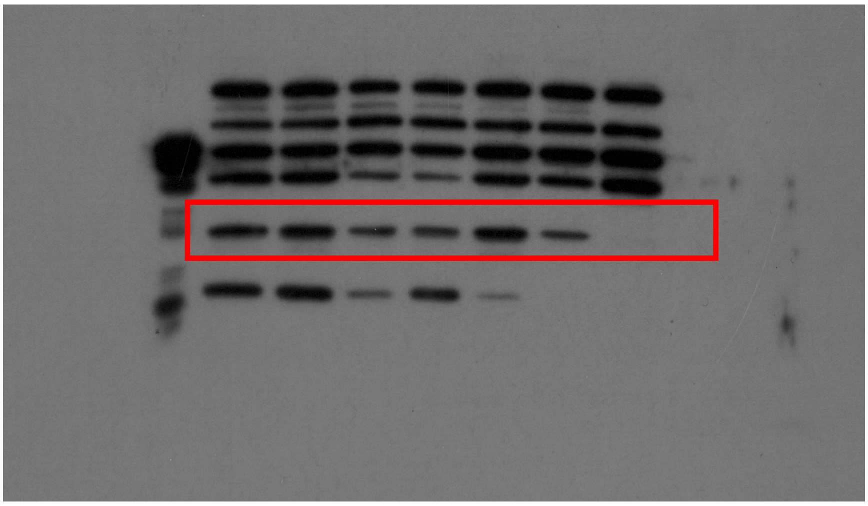

B-actin

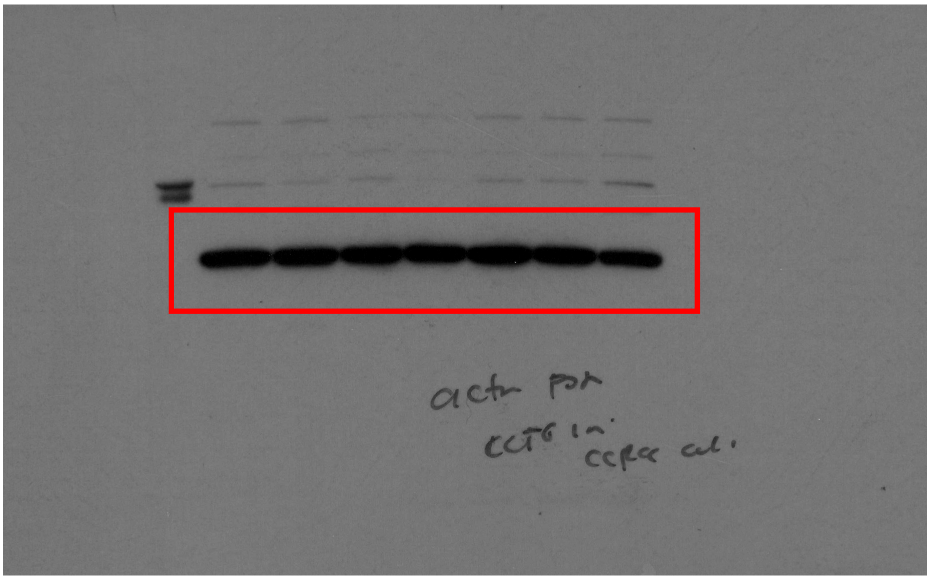

j

Full scans for Supplementary Figure 2b

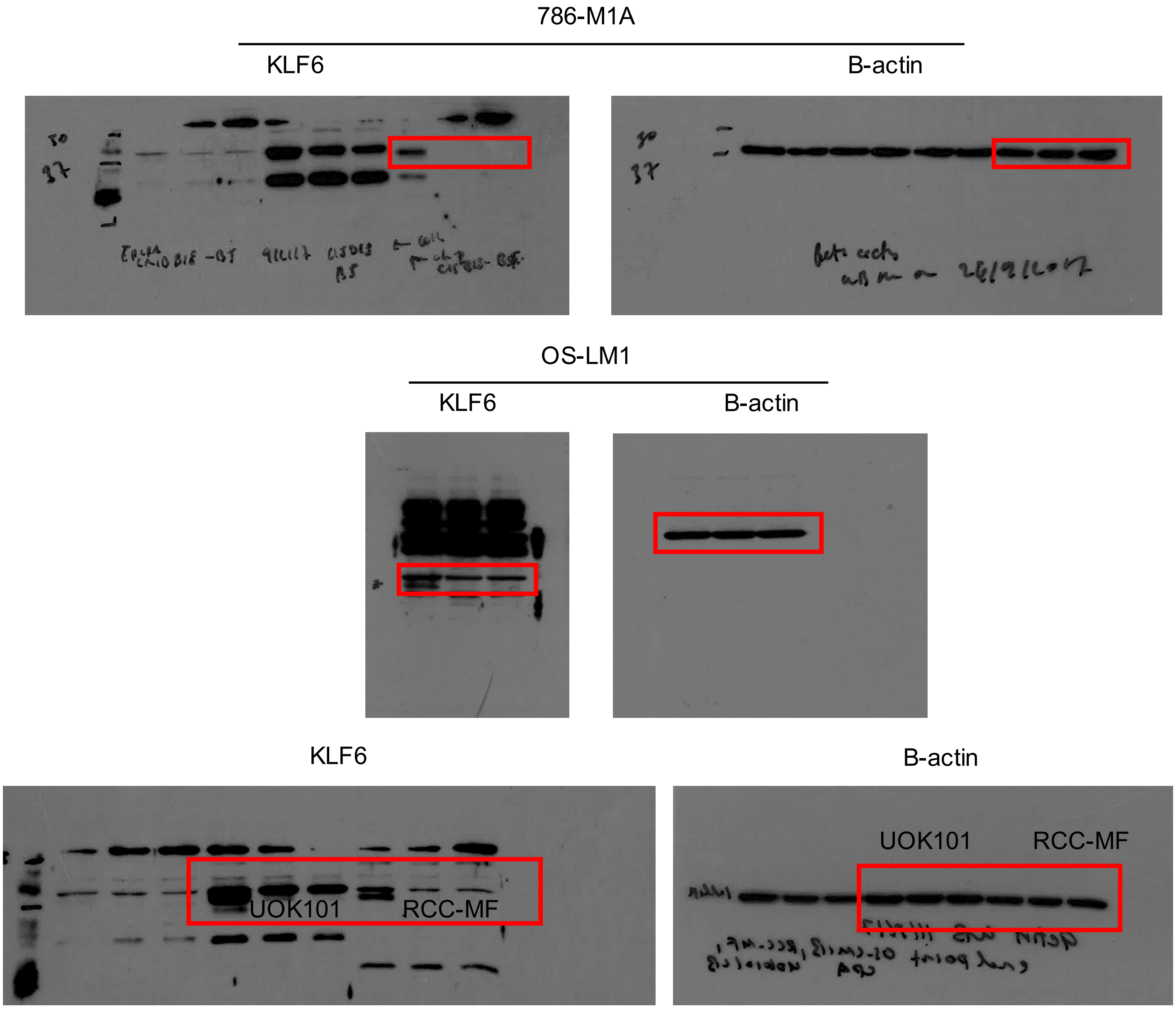

k

Full scans for Supplementary Figure 8a

SREBF2 (pre-cursor and mature)

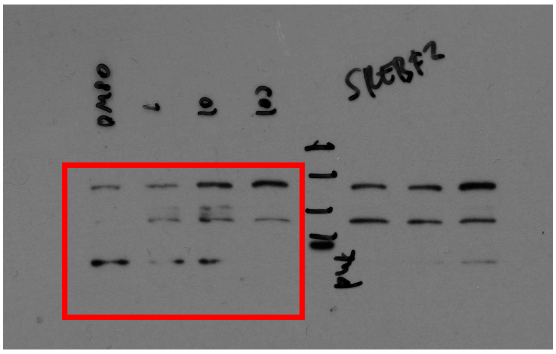

B-actin (SREBF2)

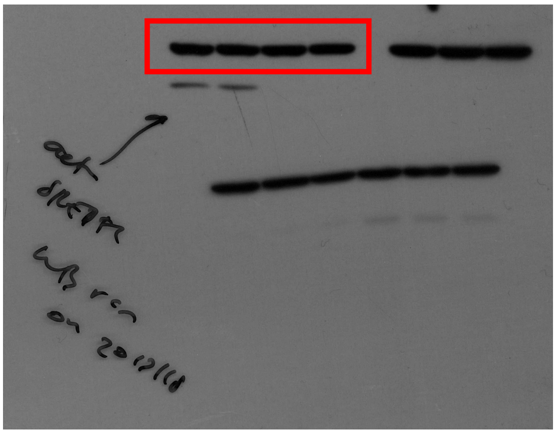

P-p70 S6 kinase

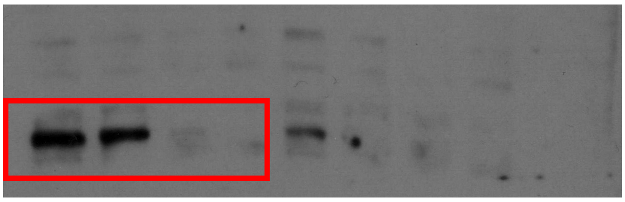

P-S6

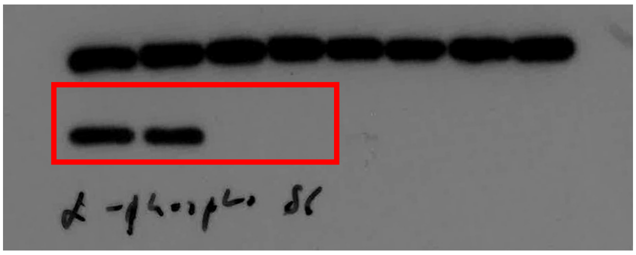

SREBF1 (pre-cursor and mature)

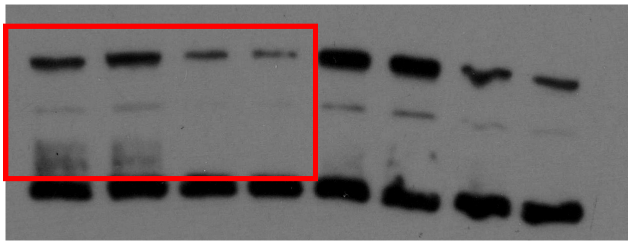

B-actin

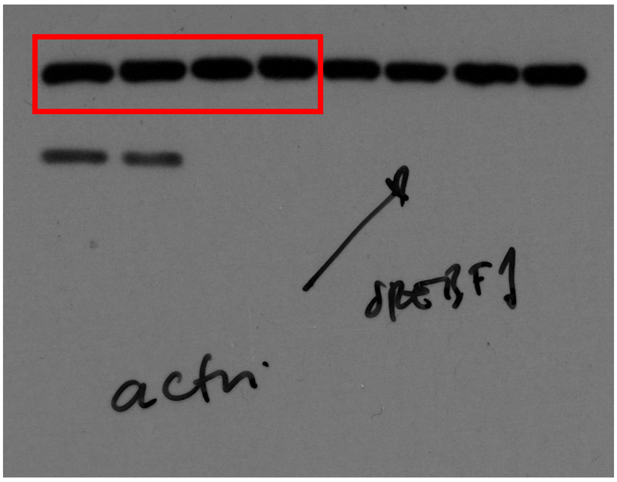

Supplementary Figure 15. Full Western blot scans. (a-k) Uncropped Western blots for all the experiments in the manuscript. Red boxes indicate the regions shown in figure panels.

Supplementary Table 1. Sequences of sgRNAs used for CRISPR-Cas9 and CRISPRi targeting.

Construct	Sequence (5'-3')
sgNTC	GAGTGTCGTC GTT GCT CC TA
sgKLF6-4	TCGCCAGGGAAG GT GC GCA G
sgKLF6-5	CCCACTTGAAAGCACACCAG
Control (Tandem)	ATCGAA GACAACAC CG GA GT G TC GT CG TT GC TCC TA GT TT TAGA GC GCA G GT G TC GCCAC CT GC GAAACACC G G GATAC G GT G C<br>GTCAATCTAGTTTTACA G TCT TC TC G
iKLF6-2	ATCGAA GACAACAC CG G CTC GCA GA GAC GCC CG GC G TT TTA GA GC GCA GG T GTC G CCACC T GC GAAACACC G GG C GAG GC GC<br>GCGGTGG GAG CG TT TTACA G TCT TC TC G
iKLF6-3	ATCGAA GACAACAC CG G CTC GCA GA GAC GCC CG GC G TT TTA GA GC GCA GG T GTC G CCACC T GC GAAACACC G GG TTA CG TT T G<br>CAGTCAGTCGTTT TACA GT CT TCT CG
iPDGFB-1	CCTCCCCCGCTGCC TCC CTA
iPDGFB-2	GGAGCCCTAGG GA GG CAG CG
iSREBP1/2 combo 1	ATCGAAGACAACACCGCAGGACA CGAAC GC G CG GA G GT TT TAGA GC GCA G GT G TC GCCAC CT GC GAAACACC G CG G GC GCAA<br>CGCAAACATGGGTT TTACA GT CT TCT CG
iSREBP1/2 combo 2	ATCGAA GACAACAC CG G T GTC CT GCC CT G GCC TCA G GT TT TAGA GC GCA G GT G TC GCCAC CT GC GAAACACC GC G CAACG CAAA<br>CATGGCGGCG TT TTACA G TCT TC TC G
iSE-1	ATCGAA GACAACAC CGA GAAT CG CT GAA GAAACG CG G TT TTA GA GC GCA GG T GTC G CCACC T GC GAAACACC GTAC T GCAC TGA<br>AGACTCGGAGTTT TACA GTC TT CTC G
iSE-2	ATCGAA GACAACAC CGA CCA GCACAAT TT G TCACC G GT TT TAGA GC G CAG G TG TC GCCA CCT GC GAAACAC CG TT GAAAAAAAAC<br>CTATCACAGTTTTACAG TCT TC TC G
iSE-3	ATCGAA GACAACAC CGA T GT G GCT CT GAAT CACCAT G TT TTA GA GC GCA GG T GTC G CCACC T GC GAAACACC GAAC GG T GAG TT<br>CCCGGTACAGTTT TACA GTC TT CTC G
iSE-4	ATCGAA GACAACAC CGA G TT G GAAAG TT GCA TG CT G GT TT TAGA GC GCA G GT G TC GCCA CCT GC GAAACAC CG TTACA CCAACA<br>GATAAATATGTTTTACAG TC TTC TC G
iSE-5	ATCGAA GACAACAC CGA TAAAG CCT G TTAT TACCAA GT TT TA GAG CG CAG G TG TC GC CACCT G CGAAACAC C GTTA GC TAAT GC T<br>GAACAGAGGTTTTACA GT CT TCT CG
Del SE 2	ATCGAA GACAACAC CG TA GAAT GTA TTA GTC TT G T GG TT TTA GA GC GCA G GT GT CG CCACC T GC GAAACACC GT G G G GTC TTA G<br>GTACCCGAAGTTTTACA G TCT TC TC G
Del SE 3	ATCGAA GACAACAC CG T G GATCCA T GAT TGA TG CAT GT TT TA GAG CG CAG G TG TC GC CACCT G CGAAACAC C GACAG CTA GT TA<br>GTGACACGAGTTT TACA GTC TT CTC G
iHIF2a binding site 1	ATCGAA GACAACAC CG TCAC TCA TCT CAGA GAAAT G GT TT TAGA GC GCA G GT G TC GCCAC CT GC GAAACACC G TCT G T GT TG CTA<br>AATCCCGAGTTTTACAG TC TTC TC G
iHIF2a binding site 2	ATCGAA GACAACAC CGA CT G GATAC G TG GA GT TAT G GT TT TAGA GC GCA G GT G TC GCCA CCT GC GAAACAC CGA TT TCAAAACCA<br>CATTTCACGTTTTACA GTC TT CTC G

Supplementary Table 2. Primer sequences.

Primers	Sequence (5'-3')
KLF6 cDNA F	ATGGACGTGCTCCCCATGTG
KLF6 cDNA R	ACATGAAGAGGCACCTCTGA
Flag KLF6 cDNA F	ATGGACTATAAGGACCACGACGGAGACTACAAGGATCATGATATTGATTACAAAGACGATGACGATAAGGGCTCCGGAGACGTGCTCCCCATGTGCAG
Flag eGFP cDNA F	ATGGACTATAAGGACCACGACGGAGACTACAAGGATCATGATATTGATTACAAAGACGATGACGATAAGGGCTCCGGAGTGAGCAAGGGCGAGGAGCT
Flag eGFP cDNA R	TTACTTGTACAGCTCGTCCA
SE del. PCR screening F	CTAGTGGACGAAGGGTTGGA
SE del. PCR screening R	AACAACCGGAATCTCCACTG
EPAS1 locus F	GGATTTTCAGACTGTTGAAT
EPAS1 locus R	CCTACAGAAGAACAGACATG
sgKLF6-4 targeted region F	P5-Read1 sequencing template-TTGCGTGCCCGGGGAGCT
sgKLF6-4 targeted region R	P7-Index-Index sequencing template-CAAGTGGGAGCTTTTGGTGT
sgKLF6-5 targeted region F	P5-Read1 sequencing template-GGAAAGTTTACACCAAAAGCT
sgKLF6-5 targeted region R	P7-Index-Index sequencing template-CTTTGGTGGAAAACATCTGA
Flag KLF6 ChIP qPCR (PDGFB) F	ATTCCGGGTAGACTTGCCAA
Flag KLF6 ChIP qPCR (PDGFB) R	GCACGGGAGATGGGGTATAA
ChIP-qPCR Ctrl 1 F	TGCGTATTAATGCTTTTCATTCC
ChIP-qPCR Ctrl 1 R	TCAATTAGTAAAGAATGATGCTTGAAA
ChIP-qPCR Ctrl 2 F	CTGCTGTTTGGGGAGCTTAC
ChIP-qPCR Ctrl 2 R	CTGCCTCCTGAATGATGACA
HIF2A ChIP qPCR F	AATCCCGAAGGCTGGTTAAT
HIF2A ChIP qPCR R	GCCCCTAGAGCAACTCTCAA
