# Supplementary material for: A KLF6-driven transcriptional network links lipid homeostasis and tumour growth in renal carcinoma
Source: Nat Commun. 2019 Mar 11;10:1152. doi: 10.1038/s41467-019-09116-x (PMC6411998; doi:10.1038/s41467-019-09116-x)
Supplement: Supplementary file 3 — Description of Additional Supplementary Files [file 41467_2019_9116_MOESM3_ESM.docx]

**Description of Additional Supplementary Files**

File Name: Supplementary Data 1

Description: Differentially expressed genes in KLF6-depleted cells.
