# Supplementary material for: A KLF6-driven transcriptional network links lipid homeostasis and tumour growth in renal carcinoma
Source: Nat Commun. 2019 Mar 11;10:1152. doi: 10.1038/s41467-019-09116-x (PMC6411998; doi:10.1038/s41467-019-09116-x)
Supplement: Supplementary file 5 — Reporting Summary [file 41467_2019_9116_MOESM5_ESM.pdf]

nature research

Corresponding author(s): Sakari Vanharanta

Last updated by author(s): 11.2.2019

Reporting Summary

Nature Research wishes to improve the reproducibility of the work that we publish. This form provides structure for consistency and transparency in reporting. For further information on Nature Research policies, see Authors & Referees and the Editorial Policy Checklist.

Statistics

For all statistical analyses, confirm that the following items are present in the figure legend, table legend, main text, or Methods section.

n/a	Confirmed	
☐	☒	The exact sample size (*n*) for each experimental group/condition, given as a discrete number and unit of measurement
☐	☒	A statement on whether measurements were taken from distinct samples or whether the same sample was measured repeatedly
☐	☒	The statistical test(s) used AND whether they are one- or two-sided<br>*Only common tests should be described solely by name; describe more complex techniques in the Methods section.*
☒	☐	A description of all covariates tested
☐	☒	A description of any assumptions or corrections, such as tests of normality and adjustment for multiple comparisons
☐	☒	A full description of the statistical parameters including central tendency (e.g. means) or other basic estimates (e.g. regression coefficient) AND variation (e.g. standard deviation) or associated estimates of uncertainty (e.g. confidence intervals)
☒	☐	For null hypothesis testing, the test statistic (e.g. *F*, *t*, *r*) with confidence intervals, effect sizes, degrees of freedom and *P* value noted<br>*Give P values as exact values whenever suitable.*
☒	☐	For Bayesian analysis, information on the choice of priors and Markov chain Monte Carlo settings
☒	☐	For hierarchical and complex designs, identification of the appropriate level for tests and full reporting of outcomes
☐	☒	Estimates of effect sizes (e.g. Cohen's *d*, Pearson's *r*), indicating how they were calculated

Our web collection on statistics for biologists contains articles on many of the points above.

Software and code

Policy information about availability of computer code

Data collection	No code was used for data collection.
Data analysis	The following programs were used for data analysis:<br>R (version 3.4.2)<br>Bowtie2 (version 2.2.2.2 and 2.3.4.3)<br>samtools (version 0.1.19)<br>MACS2 (version 2.1.0 and 2.1.1.20).<br>RSEM (version 1.2.15)<br>GSEA software (version 2-2.2.2)<br>Molecular Signature Database (MSigDB) (version 6.0) (http://www.broad.mit.edu/gsea/)<br>Ingenuity Pathway Analysis (IPA) software. (QIAGEN Inc., https://www.qiagenbioinformatics.com/products/ingenuitypathway-analysis).

For manuscripts utilizing custom algorithms or software that are central to the research but not yet described in published literature, software must be made available to editors/reviewers. We strongly encourage code deposition in a community repository (e.g. GitHub). See the Nature Research guidelines for submitting code & software for further information.

Data

Policy information about availability of data

All manuscripts must include a data availability statement. This statement should provide the following information, where applicable:

- Accession codes, unique identifiers, or web links for publicly available datasets
- A list of figures that have associated raw data
- A description of any restrictions on data availability

Previously published ChIP-seq and RNA-seq data were reanalysed from the GEO data set GSE98015 and the SRA data sets SRP007993, SRP028819, SRP097662,

SRP012412. The RNA-seq and ChIP-seq data generated during this study have been deposited to GEO under the access codes GSE115763 and GSE115749. Human RNA-seq data for different tumour types were downloaded from the TCGA data portal (http://tcga-data.nci.nih.gov/).

Field-specific reporting

Please select the one below that is the best fit for your research. If you are not sure, read the appropriate sections before making your selection.

☒ Life sciences ☐ Behavioural & social sciences ☐ Ecological, evolutionary & environmental sciences

For a reference copy of the document with all sections, see nature.com/documents/nr-reporting-summary-flat.pdf

Life sciences study design

All studies must disclose on these points even when the disclosure is negative.

Sample size	No statistical method was used to predetermine sample size.
Data exclusions	No data were excluded from the analyses.
Replication	The reproducibility of the experimental findings were verified by performing additional independent experiments (at least two) or by having several technical replicates (as described in the figure legends). Furthermore, independent experiments were also conducted in several cell lines to ensure the findings were reproducible as well as cross-checking the findings with the clinical data in the TCGA cohort. All attempts at replication were confirmed to be successful.
Randomization	Samples were not randomized.
Blinding	For histological tumour count analyses the experimental groups were blinded, for other experiments the experimental groups were not randomized or blinded.

Reporting for specific materials, systems and methods

We require information from authors about some types of materials, experimental systems and methods used in many studies. Here, indicate whether each material, system or method listed is relevant to your study. If you are not sure if a list item applies to your research, read the appropriate section before selecting a response.

Materials & experimental systems

n/a	Involved in the study	
☐	☒	Antibodies
☐	☒	Eukaryotic cell lines
☒	☐	Palaeontology
☐	☒	Animals and other organisms
☒	☐	Human research participants
☒	☐	Clinical data

Methods

n/a	Involved in the study	
☐	☒	ChIP-seq
☐	☒	Flow cytometry
☒	☐	MRI-based neuroimaging

Antibodies

Antibodies used

1. KLF6 (Santa Cruz Biotech, sc-7158, 1:1000)
2. HIF2A (Novus Biologicals, NB100-122, 1:1000),
3. VHL (BD Biosciences, 564183, 1:1000)
4. P-p70 S6-kinase (Cell Signaling Technology, Thr389, #9205, 1:1000)
5. p70-S6-kinase (Cell Signaling Technology, #9202, 1:1000)
6. P-S6 ribosomal (Cell Signaling Technology, Ser235/236, #4857, 1:3000)
7. S6 ribosomal (Cell Signaling Technology, #2317, 1:1000)
8. SREBP1 (Santa Cruz Biotech, sc-13551, 1:100)
9. SREBP2 (Santa Cruz Biotech, sc-13552, 1:100)
10. B-actin (Sigma-Aldrich, A1978, 1:20000)
11. H3K27ac (Abcam, ab4729, 5µg)
12. Monoclonal FLAG (Sigma-Aldrich, F1804, 30µg)
13. Rabbit polyclonal IgG (Abcam, ab27478, 5µg)
14. Human vimentin (Cell Signaling Technology; Cat. 5741,1:100)
15. Polyclonal goat anti-mouse IgG/HRP (Dako, P0447, 1:10000)
16. Polyclonal goat anti-rabbit IgG/HRP conjugated (Dako, P0448, 1:5000)

Validation

1. KLF6 (Santa Cruz Biotech, sc-7158, 1:1000) - antibody technical datasheet [http://datasheets.scbt.com/sc-7158.pdf]. The antibody was also validated in this study via the KLF6 knockout experiment and exogenous KLF6 reintroduction.

2. HIF2A (Novus Biologicals, NB100-122, 1:1000) - antibody technical datasheet [https://www.novusbio.com/PDFs/NB100-122.pdf].

3. VHL (BD Biosciences, 564183, 1:1000) - antibody technical datasheet [http://www.bdbiosciences.com/ds/pm/tds/564183.pdf]

4. P-p70 S6-kinase (Cell Signaling Technology, Thr389, #9205, 1:1000) - antibody technical datasheet [https://media.cellsignal.com/pdf/9205.pdf]

5. p70-S6-kinase (Cell Signaling Technology, #9202, 1:1000) - antibody technical datasheet [https://media.cellsignal.com/pdf/9202.pdf]

6. P-S6 ribosomal (Cell Signaling Technology, Ser235/236, #4857, 1:3000) -antibody technical datasheet [https://media.cellsignal.com/pdf/4857.pdf]

7. S6 ribosomal (Cell Signaling Technology, #2317, 1:1000) -antibody technical datasheet [https://media.cellsignal.com/pdf/2317.pdf]

8. SREBP1 (Santa Cruz Biotech, sc-13551, 1:100) - antibody technical datasheet [https://datasheets.scbt.com/sc-13551.pdf]

9. SREBP2 (Santa Cruz Biotech, sc-13552, 1:100) -antibody technical datasheet [https://datasheets.scbt.com/sc-13552.pdf]

10. B-actin (Sigma-Aldrich, A1978, 1:20000) - antibody technical datasheet [https://www.sigmaaldrich.com/content/dam/sigma-aldrich/docs/Sigma/Datasheet/6/a1978dat.pdf]

11. H3K27ac (Abcam, ab4729, 5µg) - antibody information [https://www.abcam.com/histone-h3-acetyl-k27-antibody-chip-grade-ab4729.html#top-0]

12. Monoclonal FLAG (Sigma-Aldrich, F1804, 30µg) - antibody technical datasheet [https://www.sigmaaldrich.com/content/dam/sigma-aldrich/docs/Sigma/Bulletin/f1804bul.pdf]

13. Rabbit polyclonal IgG (Abcam, ab27478, 5µg) - used in several publications. Examples are listed below:
- Yu et al, Cell Stem Cell (2018) PMID: 30017591
- Rodrigues et al, Cancer Discovery (2018) PMID: 29875134
- Acar et al, Nature (2015) PM1D: 26416744

14. Human vimentin (Cell Signaling Technology; Cat. 5741,1:100) - antibody technical datasheet [https://media.cellsignal.com/pdf/5741.pdf]

15. Polyclonal goat anti-mouse IgG/HRP (Dako, P0447, 1:10000) - antibody tecnical datasheet [https://www.agilent.com/cs/library/packageinsert/public/104706002.PDF]

16. Polyclonal goat anti-rabbit IgG/HRP conjugated (Dako, P0448, 1:5000) - antibody technical datasheet [https://www.agilent.com/cs/library/packageinsert/public/104707002.PDF]

Eukaryotic cell lines

Policy information about cell lines

Cell line source(s)

Human renal cancer cell lines; 786-O, 786-M1A, OS-RC2, OS-LM1, RCC-MF, were obtained from J. Massagué (MSKCC, New York, USA) in 2014. 786-M1A and OS-LM1 cells are metastatic derivatives of 786-O and OS-RC2 cells, respectively (Vanharanta et al., Nat Med. (2013) PMID: 23223005).

The UOK101 human renal cancer cell line was obtained from from Marston Linehan (The UOB Tumor Cell Line Repository, National Cancer Institute, Bethesda, MD) in 2014. The A549 lung cancer cell line was obtained from C. Martins (MRC Cancer Unit) in 2017.

Authentication

Cell lines used in this study were authenticated by short tandem repeat (STR) analysis.

Mycoplasma contamination

All cells were confirmed to be mycoplasma negative using the MycoAlertTM Mycoplasma Detection Kit (Lonza, LT07-318).

Commonly misidentified lines
(See ICLAC register)

At the time of the study, none of the cell lines used in this study were listed in the database of commonly misidentified cell lines maintained by ICLAC.

Animals and other organisms

Policy information about studies involving animals; ARRIVE guidelines recommended for reporting animal research

Laboratory animals

1. Athymic nude mice, male, 5-8 weeks old (Charles River Laboratories).
2. NOD/SCID mice, male, 5-7 weeks old (Charles River Laboratories).

Wild animals

The study did not involve wild animals.

Field-collected samples | The study did not involve samples collected from the field.

Ethics oversight | Home Office (UK) and the University of Cambridge Animal Welfare and Ethical Review Body.

Note that full information on the approval of the study protocol must also be provided in the manuscript.

ChIP-seq

Data deposition

☒ Confirm that both raw and final processed data have been deposited in a public database such as GEO.

☒ Confirm that you have deposited or provided access to graph files (e.g. BED files) for the called peaks.

Data access links
May remain private before publication.

https://www.ncbi.nlm.nih.gov/geo/query/acc.cgi?acc=GSE115749

Files in database submission

ChIP-seq_786-M1A_EV_hg38_H3K27ac.bw
ChIP-seq_786-M1A_HA-VHL_hg38_H3K27ac.bw
ChIP-seq_M1A_xenograft_1_hg38_H3K27ac.bw
ChIP-seq_M1A_xenograft_2_hg38_H3K27ac.bw
ChIP-seq_OS-LM1_EV_hg38_H3K27ac.bw
ChIP-seq_OS-LM1_HA-VHL_hg38_H3K27ac.bw
ChIP-seq_del_SE_3-15_hg38_H3K27ac.bw
ChIP-seq_del_SE_3-19_hg38_H3K27ac.bw
ChIP-seq_del_SE_3-9_hg38_H3K27ac.bw
ChIP-seq_iSE-1_hg38_H3K27ac.bw
ChIP-seq_iSE-2_hg38_H3K27ac.bw
ChIP-seq_iSE-3_hg38_H3K27ac.bw
ChIP-seq_iSE-4_hg38_H3K27ac.bw
ChIP-seq_iSE-5_hg38_H3K27ac.bw
ChIP-seq_iSE-Ctrl_hg38_H3K27ac.bw
ChIP-seq_786-M1A_FLAG-EGFP_hg38.bw
ChIP-seq_786-M1A_FLAG-KLF6_hg38.bw
ChIP-seq_786-M1A_EV_hg38_H3K27ac.fq.gz
ChIP-seq_786-M1A_HA-VHL_hg38_H3K27ac.fq.gz
ChIP-seq_M1A_xenograft_1_hg38_H3K27ac.fq.gz
ChIP-seq_M1A_xenograft_2_hg38_H3K27ac.fq.gz
ChIP-seq_OS-LM1_EV_hg38_H3K27ac.fq.gz
ChIP-seq_OS-LM1_HA-VHL_hg38_H3K27ac.fq.gz
ChIP-seq_del_SE_3-15_hg38_H3K27ac.fq.gz
ChIP-seq_del_SE_3-19_hg38_H3K27ac.fq.gz
ChIP-seq_del_SE_3-9_hg38_H3K27ac.fq.gz
ChIP-seq_iSE-1_hg38_H3K27ac.fq.gz
ChIP-seq_iSE-2_hg38_H3K27ac.fq.gz
ChIP-seq_iSE-3_hg38_H3K27ac.fq.gz
ChIP-seq_iSE-4_hg38_H3K27ac.fq.gz
ChIP-seq_iSE-5_hg38_H3K27ac.fq.gz
ChIP-seq_iSE-Ctrl_hg38_H3K27ac.fq.gz
ChIP-seq_786-M1A_FLAG-EGFP.fq.gz
ChIP-seq_786-M1A_FLAG-KLF6.fq.gz

Genome browser session
(e.g. UCSC)

NA

Methodology

Replicates | Each sample was analyzed once.

Sequencing depth

All ChIP-seq sequencing reads were 50bp single-end.

sample reads uniquelly_mapped
ChIP-seq_786-M1A_EV_hg38_H3K27ac.fq.gz 46932964 38520440
ChIP-seq_786-M1A_HA-VHL_hg38_H3K27ac.fq.gz 63296831 52195861
ChIP-seq_M1A_xenograft_1_hg38_H3K27ac.fq.gz 110044014 66673607
ChIP-seq_M1A_xenograft_2_hg38_H3K27ac.fq.gz 78964339 47931493
ChIP-seq_OS-LM1_EV_hg38_H3K27ac.fq.gz 50468835 40722181
ChIP-seq_OS-LM1_HA-VHL_hg38_H3K27ac.fq.gz 47344415 37438011
ChIP-seq_del_SE_3-15_hg38_H3K27ac.fq.gz 24297083 20438427
ChIP-seq_del_SE_3-19_hg38_H3K27ac.fq.gz 11958191 9964386
ChIP-seq_del_SE_3-9_hg38_H3K27ac.fq.gz 21628773 18113633
ChIP-seq_iSE-1_hg38_H3K27ac.fq.gz 14824438 12249928
ChIP-seq_iSE-2_hg38_H3K27ac.fq.gz 22530719 18385664
ChIP-seq_iSE-3_hg38_H3K27ac.fq.gz 14716459 11860120

ChIP-seq_iSE-4_hg38_H3K27ac.fq.gz 19464523 15664270
ChIP-seq_iSE-5_hg38_H3K27ac.fq.gz 24701460 19677651
ChIP-seq_iSE-Ctrl_hg38_H3K27ac.fq.gz 18720801 15349451
ChIP-seq_786-M1A_FLAG-EGFP.fq.gz 53525709 40830819
ChIP-seq_786-M1A_FLAG-KLF6.fq.gz 10855368 7942671

Antibodies	H3K27ac (Abcam, ab4729)<br>Monoclonal FLAG (Sigma-Aldrich, F1804)
Peak calling parameters	Raw ChIP-seq sequencing reads were aligned to hg38 using bowtie2 (2.2.2.2) and the resulting sam files were converted into sorted bam files using samtools (0.1.19). FLAG-KLF6 peaks were called using MACS2 (2.1.1.20), with FLAG-EGFP as a control.
Data quality	Initial sequencing quality was assessed by FASTQC (version 3) and sequencing read depth and mapping efficiency. Processed graph files (bw) were also inspected manually using the IGV genome browser (version 2.4.2). For FLAG-KLF6 ChIP seq peaks were called. 11531 peaks were identified at q<0.05, 9282 of which have a fold enrichment >5.
Software	Raw ChIP-seq sequencing reads were aligned to hg38 using bowtie2 (2.2.2.2) and the resulting sam files were converted into sorted bam files using samtools (0.1.19). FLAG-KLF6 peaks were called using MACS2 (2.1.1.20). The images of ChIP-seq graph files were produce using R (version 3.4.2).

Flow Cytometry

Plots

Confirm that:

☒ The axis labels state the marker and fluorochrome used (e.g. CD4-FITC).

☒ The axis scales are clearly visible. Include numbers along axes only for bottom left plot of group (a 'group' is an analysis of identical markers).

☒ All plots are contour plots with outliers or pseudocolor plots.

☒ A numerical value for number of cells or percentage (with statistics) is provided.

Methodology

Sample preparation	In brief, the cells were either lentivirally transduced with mCherry or BFP sgRNA expression vector and mixed at a 1:1 ratio. For flow cytometry analyses at the different time points, the mixed cells populations were trypsinized and directly analyzed on the instrument specified below.<br><br>Source of cells : human renal cancer cell lines
Instrument	LSR Fortessa (BD Biosciences)
Software	FlowJo software
Cell population abundance	The abundance of relevant cell population was determined based on the specific fluorescent marker expressed by the cells. The control cell population increased over times and outgrew the knockout cell population that resulted in reduced percentage/ abundance of the knockout cell population.
Gating strategy	Competitive proliferation assay gating strategy<br><br>1. FSC-A / SSC-A : to select for live cell population<br>2. FSC-H / SSC-A : to select for single cells<br>3. mCherry (561nm/610nm) and BFP (383nm/445nm) : to discriminate between the two cell populations

☒ Tick this box to confirm that a figure exemplifying the gating strategy is provided in the Supplementary Information.
